# Accelerating MRI With Longitudinally‐Informed Latent Posterior Sampling

**DOI:** 10.1002/mrm.70257

**Published:** 2026-02-22

**Authors:** Yonatan Urman, Zachary Shah, Ashwin Kumar, Bruno P. Soares, Kawin Setsompop

**Affiliations:** ^1^ Electrical Engineering Stanford University Stanford California USA; ^2^ Radiology Stanford University Stanford California USA

**Keywords:** deep learning, longitudinal MRI, reconstruction

## Abstract

**Purpose:**

To accelerate MRI acquisition by incorporating the previous scans of a subject during reconstruction. Although longitudinal imaging constitutes much of clinical MRI, leveraging previous scans is challenging due to the complex relationship between scan sessions, potentially involving substantial anatomical or pathological changes, and the lack of open‐access data sets with both longitudinal pairs and raw k‐space needed for training deep learning‐based reconstruction models.

**Methods:**

We propose a diffusion‐model‐based reconstruction framework that eliminates the need for longitudinally paired training data. During training, we treat all scan timepoints as samples from the same distribution, therefore requiring only standalone images. At inference, our framework integrates a subject's prior scan in magnitude DICOM format, which is readily available in clinical workflows, to guide reconstruction of the follow‐up. To support future development, we introduce an open‐access clinical data set containing multi‐session pairs including prior DICOMs and follow‐up k‐space.

**Results:**

Our method consistently outperforms both longitudinal and non‐longitudinal baseline reconstruction methods across various accelerated Cartesian acquisition strategies. In imaging regions highly similar to the prior scan, we observe up to 10% higher SSIM and 2 dB higher PSNR, without degradation in dissimilar areas. Compared to longitudinal reconstruction baselines, our method demonstrates robustness to varying degrees of anatomical change and misregistration.

**Conclusion:**

We demonstrate that prior scans can be effectively integrated with state‐of‐the‐art diffusion‐based reconstruction methods to improve image quality and enable greater scan acceleration, without requiring an extensive longitudinally paired training data set.

## 1 Introduction

Scan durations in MRI have decreased significantly in the past decade, notably due to advances in compressed sensing (CS) [1] and deep learning (DL)‐based reconstruction methods [2, 3]. Nonetheless, MRI remains a relatively slow imaging modality [4], limiting patient comfort and clinical throughput. At the same time, its clinical relevance continues to grow [5].

Compared to early hand‐crafted priors that exploit image sparsity [1, 6], DL‐based priors [7, 8, 9] learn data distributions, forming general regularizers over MR image features. Such methods are now approaching the achievable limit of scan acceleration attainable through learned regularizers alone, motivating the use of additional patient‐specific information in reconstruction. One well‐established example is contrast‐to‐contrast translation, where image reconstruction is conditioned on a different contrast from the same session, either synthetically [10] or through iterative data‐consistency constraints [11, 12]. Leading DL approaches for this task leverage supervised frameworks trained on multi‐contrast k‐space datasets [13, 14], of which several are publicly available [15, 16].

An underexplored regularizer of comparable potential is previous scans of the same subject. MRI is often used in longitudinal studies [17, 18, 19, 20], where patients are imaged repeatedly over time. These prior scans are typically highly correlated with newly acquired images but are rarely incorporated into the reconstruction or acquisition process of follow‐up studies.

At first glance, longitudinal reconstruction bears similarity to contrast‐to‐contrast translation, where the goal is to reconstruct one scan given another. Longitudinal reconstruction nevertheless faces a different set of practical challenges. Contrast‐to‐contrast methodologies often assume high similarity in the high‐frequency information between contrast scan‐pairs, focusing primarily on low‐frequency and predictable contrast modifications during reconstruction. However, longitudinal scans can exhibit broad‐band morphological or anatomical changes at various levels, together with scan prescription and registration variations across sessions. The conventional DL approach would be to supervise a regularizer over all possible longitudinal changes, requiring data sets of scan timepoint pairs with raw multi‐channel k‐space data for end‐to‐end training. However, to our knowledge, existing public longitudinal data sets [21, 22, 23, 24] only include magnitude images, typically in DICOM format, which are appropriate for post‐processing applications but insufficient for training complex‐valued reconstruction models [25]. Even if complex‐valued longitudinal data sets were readily available, accurately modeling all longitudinal anatomical and pathological evolutions would mandate an order of magnitude increase to the training data requirement, challenging the practicality and generalizability of conditionally supervised approaches for this problem.

With this challenge in mind, several approaches have proposed relying on prior scans only at inference. LACS [26] adds an adaptive regularization enforcing sparse differences to the prior scan, improving the standard CS reconstruction. A different approach, NERP [27], leverages the prior scan to better condition an implicit neural representation (INR) based reconstruction, showing improvement in CT with a preliminary extension to MRI. Ultimately, such approaches still underperform state‐of‐the‐art supervised DL methods agnostic of any conditioning scans. Additionally, their fidelity is sensitive to registration and structural change between scan pairs, as both methods exploit pixel‐wise similarity in their respective regularization modalities.

Hence, we observe that modeling general longitudinal regularizers is challenging both theoretically (an involute conditional distribution) and practically (inaccessible data requirement). Though longitudinal imaging is a conditional problem, we propose that, when viewed independently, the prior ${\text{x}}^{P}$ and target x reconstruction can be modeled as samples from the same marginal distribution: ${\text{x}}^{P}∼p(\text{x}),\text{x}∼p(\text{x})$. This symmetry eliminates the need to explicitly model the conditional $p(\text{x}|{\text{x}}^{P})$, utilizing unconditional approaches to instead supervise p(x) directly. With this insight, we formulate a longitudinal reconstruction framework that does not require longitudinally paired training data, but can exploit a deep general prior in conjunction with a previous scan observed at inference alone.

To accomplish this, we use a diffusion model, which has state‐of‐the‐art capability to represent an image distribution as a deep regularizer independently of the forward imaging model. We train the model on unpaired data, consisting of standalone images rather than longitudinal pairs. We specifically employ a latent diffusion model (LDM) to redirect regularization from pixel‐space to latent‐space, improving prior scan feature representation and boosting robustness to misregistration.

To leverage the prior scan, we introduce a hot‐start initialization strategy, where reconstruction begins from a noisy version of the prior scan's latent representation. While diffusion models have been used with reference images in tasks such as image editing [28, 29], these approaches typically treat the starting point as a user‐selected hyperparameter. Here, we set the initialization point adaptively based on scan similarity estimated from a fast preliminary reconstruction, balancing prior reliance with flexibility to capture image changes. This keeps reconstructions consistent with prior information while avoiding the need to explicitly model the conditional distribution.

The main contributions of this work are:

1. We propose a forward‐model‐agnostic DL framework for longitudinal MRI reconstruction that eliminates the need for paired training data. We show that incorporating the prior boosts qualitative and quantitative performance, extending the achievable neuroimaging acceleration rate for 1D variable‐density undersampling from 5–7× to 9×, and for 2D variable‐density undersampling from 20–23× to 30×, while maintaining reconstruction quality comparable to unconditional baselines at lower accelerations.
2. Compared to longitudinal reconstruction baselines, our method shows increased robustness to misregistration and varying degrees of change between scans, with a single trained model generalizing to diverse clinical applications.
3. To support future research, we curated and are actively expanding a multi‐contrast multi‐session brain MRI data set, which currently contains 232 scans from 53 subjects. The data set includes raw k‐space data with corresponding prior images, which we have publicly released for further longitudinal reconstruction benchmarking.

A preliminary version of this work appears in ISMRM 2025 [30]. Our code is available at https://github.com/SetsompopLab/LAPS.

## 2 Theory

This section begins with a brief background on LDM‐based reconstruction. We then motivate and develop a framework for hot‐starting diffusion model sampling from a given image within the model distribution, providing a basis for our proposed method.

### 2.1 Background

MRI reconstruction is commonly formulated as a maximum a posteriori problem:

(1) $${\text{x}}^{⋆}=\underset{\text{x}}{\arg\;\max}\;\log p(\text{y}|\text{x})+\log p(\text{x}).$$

Here, y=A(x)+n are undersampled measurements collected with MRI forward model A and additive Gaussian noise n. The first term in Equation (1) leads to a data‐consistency (DC) loss $\|A(\text{x})-\text{y}{\|}_{2}^{2}$, while the second term acts as a prior to regularize ill‐posed reconstructions.

#### 2.1.1 Score‐Based Diffusion Models

Learning a deep regularizer for Equation (1) requires modeling the image prior p(x). Score‐based diffusion models [31, 32] are currently state‐of‐the‐art for this task. They define a forward process that gradually perturbs data into Gaussian noise, then learn to reverse it. Generation is performed by solving the reverse‐time SDE:

(2) $$d{\text{x}}_{t}=\left[f(t){\text{x}}_{t}-g^{2}(t)\nabla_{{\text{x}}_{t}}\log p({\text{x}}_{t})\right]dt+g(t)d{\text{w}}_{t}$$

where f(t) and g(t) are time‐dependent drift and diffusion coefficients, ${\text{w}}_{t}$ is a Wiener process, and the score function $\nabla_{\text{x}}\log p_{t}(\text{x})$ is approximated by a neural network ${\text{s}}_{\theta }^{⋆}({\text{x}}_{t},t)$ trained via denoising score matching [33]. Notably, training is agnostic to A; that is, a single model can be broadly applied to various reconstruction tasks.

In DDPM [31] the reverse process is typically discretized into T=1000 steps, forming noisy intermediates ${\left\{{\text{x}}_{t}\right\}}_{t=0}^{T}$, where ${\text{x}}_{0}∼p(\text{x})$. Variants like DDIM [34] accelerate high‐quality sampling to as few as 50 steps.

#### 2.1.2 Latent Diffusion Models

Modeling p(x) in high‐dimensional image space (i.e., total number of voxels) requires learning feature‐based data representations alongside the underlying distribution, which is intensive in both data and compute. LDMs [35] address this by first training a Variational Autoencoder (VAE) [36] to map images into a compact latent space p(z) using an encoder‐decoder pair (ℰ,𝒟). For 2D complex MRI images of shape N×M:

(3) $$\begin{array}{ll} & ℰ:\text{x}\in {\mathbb{C}}^{N\times M}\to \text{z}\in {\mathbb{R}}^{\frac{N}{K}\times \frac{M}{K}\times C} \end{array}$$

(4) $$\begin{array}{ll} & 𝒟:\text{z}\in {\mathbb{R}}^{\frac{N}{K}\times \frac{M}{K}\times C}\to \text{x}\in {\mathbb{C}}^{N\times M} \end{array}$$

where K is a spatial down‐sampling factor. A diffusion model is then trained in this latent space, and samples are decoded back as images via ${\text{x}}_{0}=𝒟({\text{z}}_{0})$. This setup enables learning in a lower dimensional, semantically meaningful space, accelerating training and improving image synthesis.

#### 2.1.3 Posterior Sampling: Solving Inverse Problems With Diffusion Models

To reconstruct images given a diffusion model and y, we use posterior sampling [9, 37], which aims to draw samples from p(x|y). Since diffusion sampling relies on the score function (Equation (2)), Bayes' rule gives:

(5) $$\nabla_{{\text{x}}_{t}}\log p({\text{x}}_{t}|\text{y})=\underset{\text{Prior Score}}{\underbrace{\nabla_{{\text{x}}_{t}}\log p({\text{x}}_{t})}}+\underset{\text{Likelihood Score}}{\underbrace{\nabla_{{\text{x}}_{t}}\log p(\text{y}|{\text{x}}_{t})}}$$

The prior score is provided by a pre‐trained diffusion model, and the likelihood score enforces DC with y.

As y depends on the denoised ${\text{x}}_{0}$, not directly on ${\text{x}}_{t}$, Diffusion Posterior Sampling (DPS) [38] estimates ${\text{x}}_{0}\approx 𝔼[{\text{x}}_{0}|{\text{x}}_{t}]$ and adds a DC gradient with step‐size $\zeta_{t}$:

(6a) $$\begin{array}{ll} {\text{x}}_{t-1} & \leftarrow \text{Diffusion‐Step}({\text{x}}_{t},{\text{s}}_{\theta }^{⋆},t) \end{array}$$

(6b) $$\begin{array}{ll} {\text{x}}_{t-1} & \leftarrow {\text{x}}_{t-1}-\zeta_{t}\nabla_{{\text{x}}_{t}}\|\text{y}-A(𝔼[{\text{x}}_{0}|{\text{x}}_{t}]){\|}_{2}^{2} \end{array}$$

The expectation is computed via Tweedie's formula [39, 40]:

(7) $$𝔼[{\text{x}}_{0}|{\text{x}}_{t}]=\frac{1}{\sqrt{{\bar{\alpha }}_{t}}}\left({\text{x}}_{t}-\sqrt{1-{\bar{\alpha }}_{t}}{\text{s}}_{\theta }^{⋆}({\text{x}}_{t},t)\right)$$

Here, ${\bar{\alpha }}_{t}\;\in [0,1]$ is the cumulative noise factor which decreases monotonically with t [31].

In Latent‐DPS (LDPS) [41, 42], this update is applied in latent space, and the decoder 𝒟 is incorporated into the DC term in Equation (6b):

(8) $${\text{z}}_{t-1}\leftarrow {\text{z}}_{t-1}-\zeta_{t}\nabla_{{\text{z}}_{t}}\|\text{y}-A(𝒟(𝔼[{\text{z}}_{0}|{\text{z}}_{t}])){\|}_{2}^{2}$$

Though introducing the overhead of backpropagation through 𝒟, LDPS enables efficient and flexible reconstruction from compressed representations using LDMs.

### 2.2 Longitudinally Accelerated Posterior Sampling

At a high level, we propose leveraging the prior scan to hot‐start LDPS at an intermediate timestep $t_{p}<T$. In the following sections, we motivate this strategy, describe how to choose $t_{p}$, and highlight the benefit of latent space for this prior injection.

We consider longitudinal reconstruction with a pre‐trained complex LDM. This model defines a latent distribution at timestep t=0, denoted $p_{0}(\text{z})$, assumed to approximate the true latent‐image distribution. The posterior conditional distribution given measurements y is $p_{0}(\text{z}|\text{y})$. We denote the probability of sampling z, when starting the diffusion process from timestep t with latent ${\text{z}}_{t}$, as $p_{0}(\text{z}|{\text{z}}_{t})$, with unconditional sampling given by $p_{0}(\text{z}|{\text{z}}_{T})=p_{0}(\text{z})$.

Here, we assume access to one prior scan, ${\text{x}}^{P}$, which shares contrast characteristics with an unknown follow‐up scan, ${\text{x}}^{⋆}$, with latents ${\text{z}}^{P}=ℰ({\text{x}}^{P})$ and ${\text{z}}^{⋆}=ℰ({\text{x}}^{⋆})$, respectively. In this section, we assume ${\text{x}}^{P}$ and ${\text{x}}^{⋆}$ are phase‐aligned for simplicity, as ℰ takes a complex input. In practice, ${\text{x}}^{P}$ is magnitude‐only; this mismatch will be addressed with a phase initialization step in Section 2.2.2.

#### 2.2.1 Initialized Sampling in Latent Space

Conventional diffusion sampling begins from pure Gaussian noise, ${\text{z}}_{T}∼𝒩(0,I)$, allowing unconditional sampling from the target distribution $p_{0}(\text{z})$. Posterior sampling given y constrains the generative process toward the desired ${\text{z}}^{⋆}$. Ideally, ${\text{z}}^{⋆}={\arg\;\max}_{\text{z}}p_{0}(\text{z}|\text{y})$. But, as A becomes more ill‐posed, the space of $p_{0}(\text{z})$ consistent with y expands, hindering the exact recovery of ${\text{z}}^{⋆}$.

To further constrain the probable output space of posterior sampling, we turn to the longitudinal setting. Assuming that ${\text{x}}^{P}$ and ${\text{x}}^{⋆}$ share similar contrast and global structure, there is high likelihood that the scan pair share similar latent representations in $p_{0}(\text{z})$, suggesting that ${\text{z}}^{P}$ could inform the sampling process of ${\text{z}}^{⋆}$ via some soft constraint. Without explicitly learning this conditional relationship on a paired dataset, we propose to use ${\text{z}}^{P}$ only to initialize sampling. The key question then becomes how to do this effectively without over‐biasing the reconstruction to ${\text{x}}^{P}$.

Consider first an ideal scenario: if ${\text{z}}^{⋆}$ was accessible, we could project it to any intermediate t<T using [31]:

(9) $${\text{z}}_{t}^{⋆}=\sqrt{{\bar{\alpha }}_{t}}{\text{z}}^{⋆}+\sqrt{1-{\bar{\alpha }}_{t}}\epsilon ,\;\epsilon ∼𝒩(0,I).$$

Sampling from time t<T initialized with ${\text{z}}_{t}^{⋆}$ would inherently converge to ${\text{z}}^{⋆}$ with higher probability than sampling from T initiated by random noise ${\text{z}}_{T}$, as:

(10) $$p_{0}({\text{z}}_{0}={\text{z}}^{⋆}|{\text{z}}_{t}={\text{z}}_{t}^{⋆})>p_{0}({\text{z}}_{0}={\text{z}}^{⋆}|{\text{z}}_{T})=p_{0}({\text{z}}^{⋆}).$$

ALGORITHM 1 **LAPS.**

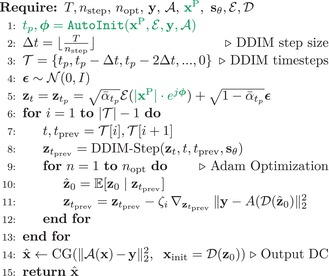

ALGORITHM 2 **CAPS.**

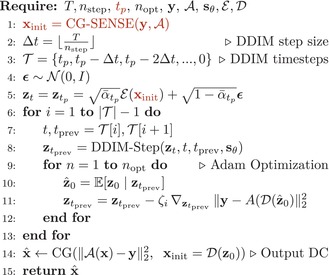

ALGORITHM 3 **AutoInit.**

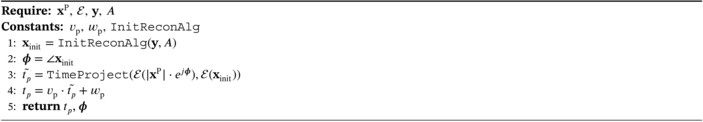

Building upon this intuition, we propose a similar initialization strategy using the prior. If we project ${\text{z}}^{P}$ to an intermediate timestep:

(11) $${\text{z}}_{t}^{P}=\sqrt{{\bar{\alpha }}_{t}}{\text{z}}^{P}+\sqrt{1-{\bar{\alpha }}_{t}}\epsilon ,\;\epsilon ∼𝒩(0,I),$$

then t should be selected to maximize the likelihood of sampling ${\text{z}}^{⋆}$, which we denote as $t_{p}$:

(12) $$t_{p}={\arg\;\max}_{t}p_{0}({\text{z}}^{⋆}|{\text{z}}_{t}={\text{z}}_{t}^{P}).$$

In the worst case, setting $t_{p}=T$ reverts to traditional posterior sampling, maintaining the reconstruction quality of the unconditional case. However, if an intermediate $t_{p}<T$ improves the likelihood in Equation (12), ${\text{z}}_{t}^{P}$ becomes a better initialization for sampling towards ${\text{z}}^{⋆}$, potentially enhancing reconstruction performance.

To select $t_{p}$ for a specific target‐prior pair, we derive the following expression for Equation (12) in Appendix A, assuming a Gaussian target latent space ${\text{z}}_{0}∼𝒩(0,I)$:

(13) $$\begin{array}{ll} t_{p}^{⋆} & ={\arg\;\max}_{t}\left\{\gamma_{t}+\frac{{\bar{\alpha }}_{t}}{2}\left[\frac{\|{\text{z}}^{P}{\|}_{2}^{2}}{2-{\bar{\alpha }}_{t}}-\frac{\|{\text{z}}^{⋆}-{\text{z}}^{P}{\|}_{2}^{2}}{2(1-{\bar{\alpha }}_{t})}\right]\right\} \end{array}$$

(14) $$\begin{array}{ll} & :=\text{TimeProject}({\text{z}}^{P},{\text{z}}^{⋆}) \end{array}$$

Equation (13) analytically relates the optimal initialization to a similarity measure between the scan pair, $\|{\text{z}}^{⋆}-{\text{z}}^{P}{\|}_{2}^{2}$, alongside noise parameters $\gamma_{t}$ and ${\bar{\alpha }}_{t}$.

Intuitively, TimeProject selects a timestep (i.e., noise level) matched to the latent discrepancy between scans. Under‐selecting $t_{p}$ may overfit to the prior, while selection close to T may discard useful prior information. Supplementary Figure S1 illustrates this trade‐off: similar scans favor smaller $t_{p}^{⋆}$ to better leverage the prior, while dissimilar scans require larger $t_{p}^{⋆}$ for flexibility. Figure S1 also shows Equation (13) is concave in t for fixed ${\text{z}}^{P}$ and ${\text{z}}^{⋆}$, implying optimality of an intermediate value.

In practice, we do not have access to ${\text{z}}^{⋆}$ at inference, and the Gaussianity assumption on ${\text{z}}_{0}$ does not necessarily hold, thus we cannot directly apply Equation (13). In the next section, we thus propose an approximation for $t_{p}$, which is integrated into our full reconstruction method.

#### 2.2.2 Finding a Good Initialization for LDPS

Hot‐starting latent posterior sampling with the prior scan has two major hurdles:

1. Prior scans are not phase‐aligned (in our case, we assume access to magnitude‐only ${\text{x}}^{P}$'s).
2. The projection timepoint $t_{p}$ must consider the level of change between scans, including mis‐registration and general scan variability.

To address these, we introduce AutoInit (summarized in Algorithm 3), which uses a preliminary reconstruction ${\text{x}}_{\text{init}}$ generated through some fast reconstruction algorithm InitReconAlg(·) to estimate both a projection timepoint $t_{p}$ and a phase map $\phi \in {\mathbb{R}}^{N\times M}$. While any method can be used for InitReconAlg, our chosen approach leverages a fast query of the trained LDM agnostic of ${\text{x}}^{P}$, further described in Section 3.2.1.

To address (1), the initializing phase is extracted as $\phi =∠{\text{x}}_{\text{init}}$. After registration to ${\text{x}}_{\text{init}}$, the prior can then be updated as $|{\text{x}}^{P}|e^{j\phi }$. For (2), we use ${\text{z}}_{\text{init}}=ℰ({\text{x}}_{\text{init}})$ as a proxy for ${\text{z}}^{⋆}$ when estimating $t_{p}$ via Equation (14), denoting

(15) $${\tilde{t}}_{p}=\text{TimeProject}({\text{z}}^{P},{\text{z}}_{\text{init}}).$$

To account for the simplifying assumptions of Equation (15), we then calibrate this estimate using a small paired validation set $D_{v}={\left\{({\text{x}}_{i}^{⋆},{\text{x}}_{i}^{P},{\text{y}}_{i})\right\}}_{i=1}^{N_{v}}$. For each example, we run reconstructions across a grid of $t_{p}$ values from T down to 0, selecting the empirically optimal $t_{p}^{⋆,\text{obs}}$ based on reconstruction metrics. We also compute ${\tilde{t}}_{p}$ from ${\text{x}}_{\text{init}}$ via Equation (15). We then fit affine parameters ($v_{p}$, $w_{p}$) such that $t_{p}=v_{p}\cdot {\tilde{t}}_{p}+w_{p}\approx t_{p}^{⋆,\text{obs}}$. Supplementary Figure S2 describes the implementation details of this calibration, verifying that $t_{p}^{⋆,\text{obs}}$ well approximates ${\tilde{t}}_{p}$.

## 3 Methods

Building on the framework introduced in the previous sections, we now present our complete reconstruction method. We first pretrain an LDM on unpaired complex images. Then, we use the prior‐based initialization scheme introduced in Section 2.2 in conjunction with a modified LDPS [42] approach. Specifically, instead of using a single latent DC gradient step as in Equation (8), we use $n_{\text{opt}}>1$ steps. This iterative correction improves performance by perturbing each $z_{t}$ closer to the manifold of data consistent latents. To control the update size adaptively, we use an Adam optimizer [43].

To enable faster reconstruction with fewer diffusion steps, we replace the standard DDPM sampling in LDPS with DDIM. Finally, since the learned decoder 𝒟 is lossy, we include additional image‐domain DC steps at the output of latent sampling.

**FIGURE 1:**
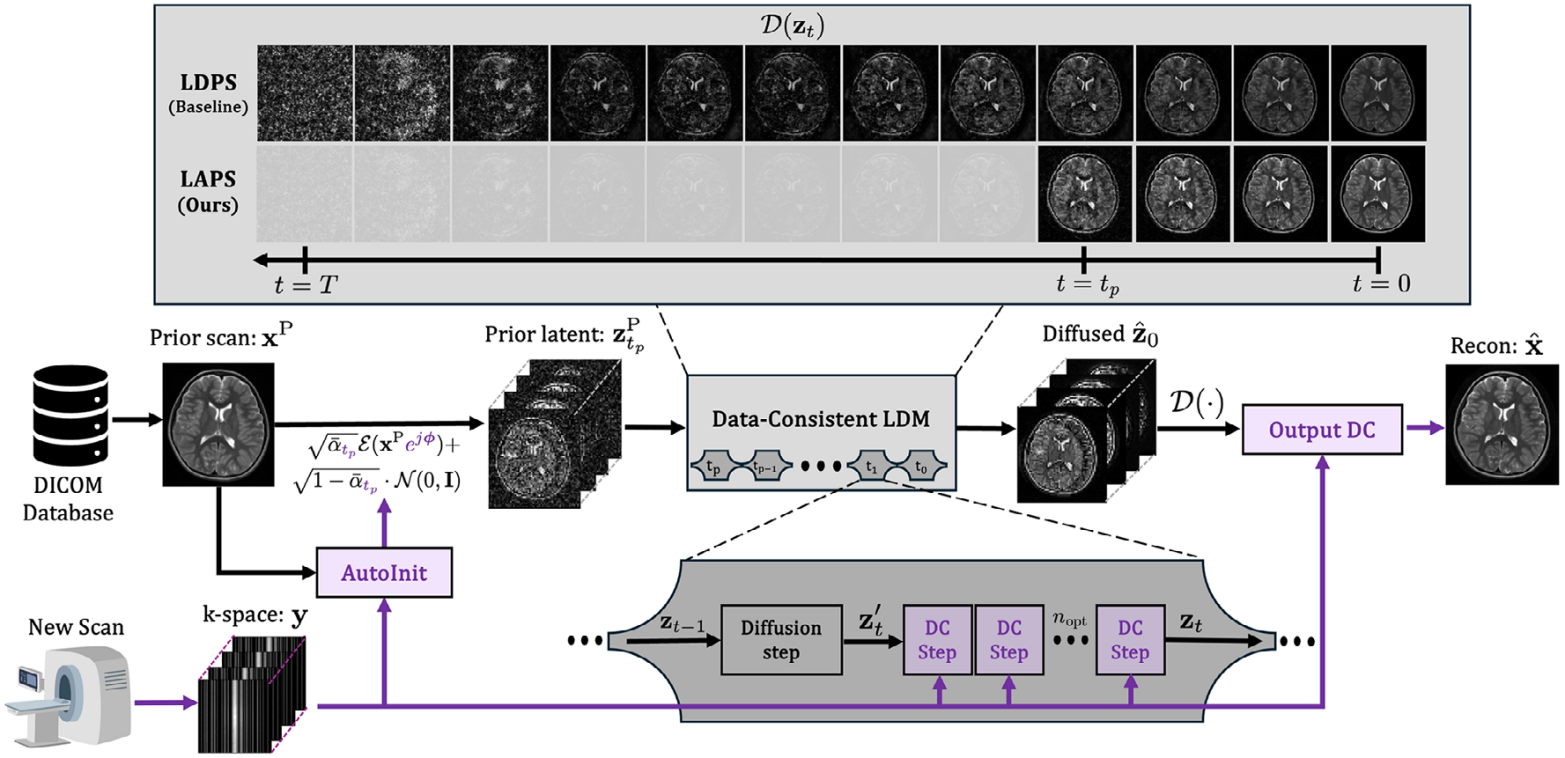
Illustration of our proposed method, LAPS. From left to right: We begin with undersampled k‐space from a new scan and retrieve the corresponding prior DICOM scan of the subject. Given these, we run a fast initial reconstruction through AutoInit, estimating an initial phase ϕ and optimal projection point $t_{p}$ to hot‐start the diffusion process with the prior. We encode the phase‐modulated prior and project it to $t_{p}$, which provides the initialization for our proposed LDM reconstruction that also incorporates the newly acquired k‐space data. Finally, the decoded output of the diffusion process is passed through a few additional DC steps, refining the output image. Compared to LDPS (a previous method), LAPS starts the diffusion process much earlier than T via AutoInit, runs $n_{\text{opt}}$ DC steps per iteration rather than 1, and enforces additional DC at the output.

We name this reconstruction method **L**ongitudinally **A**ccelerated **P**osterior **S**ampling (LAPS), illustrated in Figure 1 and summarized in Algorithm 1. To improve on a single random initialization, our final reconstruction averages over $n_{\text{avg}}$ samples generated in parallel, known in diffusion literature to further improve image quality [9].

As a variant of our reconstruction framework that does not use the prior scan, we introduce **C**onjugate Gradient‐Initialized **A**ccelerated **P**osterior **S**ampling (CAPS). Instead of the prior, it initializes latent sampling from a CG reconstruction projected to a fixed $t_{p}$, then follows the same steps as LAPS (see Algorithm 2 for direct comparisons). CAPS serves as a strong prior‐free baseline, matching or outperforming other LDM‐based methods such as PLDS [42] in our experiments, while decreasing reconstruction times (see Supplementary Figure S3). The comparison between CAPS and LAPS isolates the added benefit of longitudinal information.

### 3.1 Stanford Longitudinally Accelerated MRI (SLAM) Data Set

A major barrier to developing and evaluating longitudinal MRI reconstruction methods is the lack of available datasets suited for the task. To fill this gap, we collaborated with Stanford Hospital to curate a dataset containing prior DICOM scans and follow‐up scans with k‐space across multiple neuroimaging protocols. We call this the **S**tanford **L**ongitudinally **A**ccelerated **M**RI (SLAM) dataset, which spans a variety of scan types, contrasts, intervals, and pathologies.

The LAPS framework has different data requirements for training and testing: training the LDM requires a data set of complex‐valued reconstructions (not longitudinally paired), whereas evaluation requires paired data consisting of a prior DICOM scan and the raw k‐space of a follow‐up scan. SLAM supports both: it provides raw k‐space with corresponding complex reconstructions for LDM training, and many scans also include prior DICOMs for longitudinal evaluation.

In this paper, we present results based on SLAM as of writing. Table 1 summarizes its size, and Table 2 lists longitudinal descriptors through elapsed intervals between scans and radiologist‐graded change levels, demonstrating the dataset's range in longitudinal variability. Additional breakdowns by protocol and contrast are provided in Supplementary Table S1. All data were acquired on 3T GE systems, split by subject into train and test sets.

**TABLE 1:** Summary statistics of scans in SLAM data set, with the training and testing dataset partitions. The *Paired type* field classifies how much data is paired. *All* indicates the total number of scans with raw k‐space data, and *Pair* specifies scans with a matching prior scan. Paired typing used per partition indicated in bold.

Category	Total	Train		Test
Paired Type	All	All	Pair	Pair
**Subjects**	53	47	25	6
**Scanners**	13	13	7	3
**Scans**	232	209	81	21
**2D slices**	34970	32600	9968	1970

**TABLE 2:** Statistics of longitudinal change in SLAM data set, listed by subject.

Interval	Train	Test
(a) Time between scan sessions		
< **1 mo.**	2	1
**1–4 mo.**	11	3
**4–8 mo.**	9	2
> **8 mo.**	5	—
**N/A**	20	—
(b) Radiologist change ratings		
**Low**	12	2
**Med.**	8	3
**High**	7	1
**N/A**	20	—

Acquired in typical clinical settings, most scans were collected with 24–48‐channel head coils and mild undersampling (R=2 for 2D acquisitions; R=2–5 for 3D cases, of which a subset had variable density sampling for CS reconstruction). To get complex‐valued images of the follow‐up k‐space for network training, we implemented our own reconstruction pipeline on the raw multi‐coil k‐space. First, we trimmed the raw k‐space to a maximum matrix size of 256×256 for consistency and network compatibility, followed by SVD‐based coil compression to retain 95% of the SVD energy along the coil dimension. We then performed an L1‐wavelet CS [1] reconstruction to get complex‐valued images, leveraging sensitivity maps estimated from fully sampled center regions via ESPIRiT [44]. We finally excluded scans with visible artifacts (e.g., motion, low SNR) or poor reconstruction quality.

For this preliminary study, we focus on 2D reconstructions: as such, 3D acquisitions were converted to 2D slices via an inverse FFT along the readout dimension. For each slice, we saved the full k‐space, acquisition mask, and our reconstruction, enabling further undersampling retrospectively (see examples in Supplementary Figure S4).

For scans with prior DICOM images, we registered across sessions using an affine rigid transformation provided by the Advanced Normalization Tools (ANTs) library [45, 46]. Though longitudinal reconstruction methods depend on possibly mis‐registered previous scans, we assume a rough affine registration can first be completed with an initial reconstruction, which we already require with LAPS for phase initialization. Unless otherwise noted, we assume scans are registered in our analysis, but we additionally compare performance under controlled mis‐registration settings.

We are still actively collecting new data for SLAM. We plan to expand our public release to enable training of more complex longitudinal frameworks in the future.

### 3.2 Model Evaluation

To evaluate our proposed method, we designed several experiments involving various longitudinal and undersampling conditions. The following section describes implementation details of our method and selected baselines, which includes both classical and learning‐based approaches, with and without longitudinal priors.

#### 3.2.1 LAPS: Our Proposed Method

LAPS is an LDM‐based reconstruction method, hence it requires two components (Section 2.1.2): a VAE defining the latent space and a diffusion model operating within it. We first trained the VAE, then trained the diffusion model on the resulting latent representation.

As the VAE backbone, we adapted Med‐VAE [47] to complex‐valued MRI. Since the original model operated on magnitude images, we modified its first layer to accept two channels by replicating its weights and stacking real and imaginary inputs. We set K=4 and C=4 in Equation (3).

With the VAE trained, we then trained the diffusion model (${\text{s}}_{\theta }$). We developed a modified denoising architecture derived from the UNet of Stable Diffusion (SD) 1.5 [35], a high‐resolution text‐to‐image LDM. To remove text conditioning, which LAPS does not use, we replaced all cross‐attention layers with self‐attention. We then fine‐tuned the modified UNet on the latent representations produced by Med‐VAE.

Both Med‐VAE and the diffusion model were trained on the same dataset: all follow‐up reconstructions in the SLAM training set (32,600 complex‐valued slices), FastMRI brain [3] (13,000 $T_{2}$ and 5300 $T_{1}$ slices), and about 10,000 magnitude DICOMs of prior SLAM scans. To use DICOMs for complex‐valued training, we added a random quadratic spatially varying phase to approximate RF phase profiles in coil‐combined images. Each model was trained for 4 days on 5 NVIDIA A6000 GPUs.

For InitReconAlg, we used our same reconstruction network with a highly accelerated CAPS reconstruction, fixing $t_{p}=200$, $n_{\text{opt}}=5$, $n_{\text{step}}=80$, and $n_{\text{avg}}=1$, which takes approximately 1/10 as long as the full LAPS reconstruction. Using this ${\text{x}}_{\text{init}}$, we calibrated the $t_{p}$ affine transform used in AutoInit, yielding $v_{p}=1.55,w_{p}=-350$ (derived from the calibration in Supplementary Figure S2).

For the remaining hyperparameters, we found $n_{\text{opt}}=10$ to perform best with $n_{\text{step}}=100$ total DDIM steps (see Supplementary Figure S5), followed by 6 CG steps for the output DC. We report final reconstructions as the average over $n_{\text{avg}}=4$ samples.

#### 3.2.2 Baseline Reconstruction Methods

First, we compare our approach with other longitudinal reconstruction methods. For fair comparison to LAPS, we only consider unsupervised methods which do not require longitudinally paired training data sets, relying on ${\text{x}}^{P}$ only at inference:

1. **LACS** [26]: A compressed sensing method that promotes wavelet sparsity and similarity to ${\text{x}}^{P}$ by solving
(16) $$\begin{array}{ll} \underset{\text{x}}{\arg\;\max}\|A\text{x}-\text{y}{\|}_{2}^{2} & +\lambda_{1}\|{\text{W}}_{1}\Psi \text{x}{\|}_{1}\\ & +\lambda_{2}\|{\text{W}}_{2}(\text{x}-{\text{x}}^{P}){\|}_{1}. \end{array}$$
  Here, Ψ is a wavelet transform and ${\text{W}}_{1},{\text{W}}_{2}$ are spatially adaptive weights updated after each iteration.
2. **NERP** [27]: Models ${\text{x}}^{P}$ using an INR that is subsequently fine‐tuned via DC with the follow‐up k‐space. To support complex‐valued data, we first estimate an initial phase from a CG‐SENSE reconstruction and apply this phase during INR pre‐training, analogous to the fast phase initialization in LAPS. This produces a complex INR prior that is better aligned with the follow‐up data, improving stability during the DC‐based fine‐tuning stage.

To ablate the utility of using longitudinal information in reconstruction, we compare with other DL methods which are agnostic of prior scans:

1. **MODL** [7]: A model‐based DL method unrolling CG DC with CNN‐based denoising, trained end‐to‐end per acceleration rate with supervised references. Since MODL requires paired k‐space and reference images, we train it only on the SLAM partition of follow‐up scans that provides both, otherwise using same hyper‐parameters, training strategy, and network structure of the original paper.
2. **AdaDiff** [48]: An image‐space diffusion‐based reconstruction method, implemented using the official open‐source code and trained on the same data set and compute as LAPS for 14 days. AdaDiff benchmarks our LDM approach against a state‐of‐the‐art image‐domain approach.
3. **Conjugate Gradient‐Initialized LDPS (CAPS)**: Our non‐longitudinal variant of LAPS, described in Section 3. Except for fixing $t_{p}=200$, all hyperparameters match those used in LAPS.

#### 3.2.3 Metrics

We assess performance of our method and baselines on the SLAM test partition (21 scans from 6 subjects, per Table 1). We evaluate reconstructions on retrospectively undersampled k‐space at various rates. 2D or 3D acquisitions with a single undersampling dimension are evaluated with 1D retrospective undersampling, whereas 3D acquisitions with two undersampling dimensions are evaluated only with 2D retrospective undersampling. For scans that were fully sampled in the original acquisition, we generate both 1D and 2D retrospective undersampling patterns, allowing evaluation under both cases. Undersampling followed a variable density sampling pattern selecting only from the set of lines or points acquired, with an iterative re‐sampling scheme to minimize large gaps in the generated masks (see Supplementary Figure S4 for example masks in each case). All volumetric reconstructions are completed slice by slice, fitting on a single A6000 GPU. For evaluation, we include only 15 evenly spaced slices per volume to avoid biasing to scans with more slices, resulting in evaluation over 225 slices with 1D undersampling and 150 slices for 2D undersampling. All retrospective coil‐compressed k‐space retains a fully sampled center, included in the reported undersampling rate R, used for ESPIRiT calibration.

To assess global reconstruction quality, we compute PSNR and SSIM over all test set slices. Taken standalone, these metrics can be misleading in longitudinal reconstruction, where much of the anatomy may remain unchanged, potentially masking errors in regions with differences. To address this, we also evaluate performance in local image regions classified by similarity between scan timepoints. Per prior‐target scan pair, we divide pixel space into 32×32 patches with 50% overlap and compute cosine similarity between corresponding patch pairs. We then aggregate all patches across the test set, bin by cosine similarity percentile, and report average reconstruction PSNR and SSIM over all patches per bin.

## 4 Results

### 4.1 Performance Versus Acceleration

**FIGURE 2:**
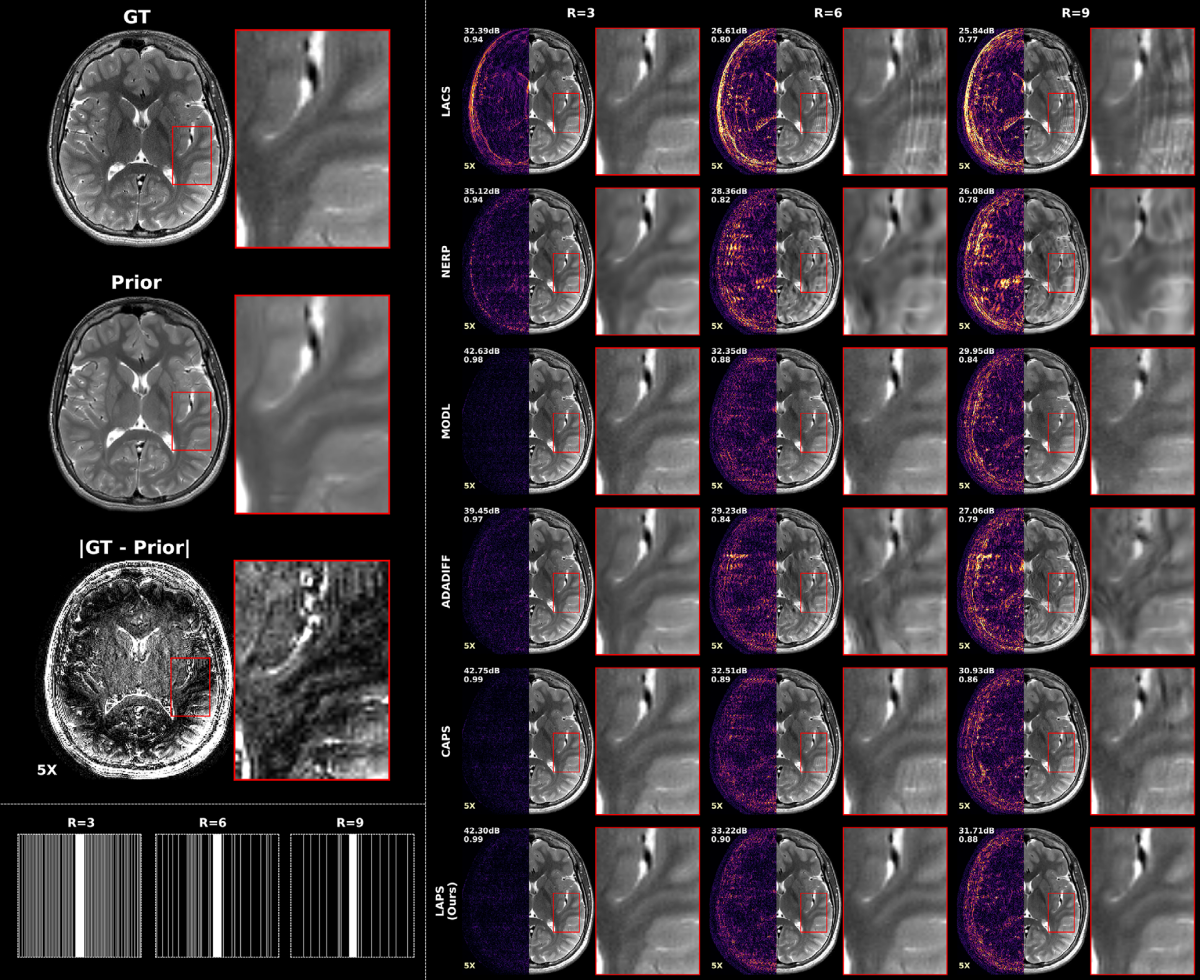
Comparison of reconstruction methods at varying 1D accelerations in a case with minimal longitudinal change. Axial $T_{2}$‐weighted images at the level of the basal ganglia and insula in a patient with an incidentally discovered sub‐centimeter cerebellar lesion (not shown), presumed to be a low‐grade tumor. The lesion and other brain regions remained stable between the two exams performed 6 months apart. The left column shows, from top to bottom, the target (ground truth) image, the prior scan, and the amplified difference between them. Underneath these are example undersampling masks used for reconstruction at acceleration rates of R=3, 6, and 9. The right‐side grid presents reconstructions from different methods at each acceleration rate, with our proposed method (LAPS) shown in the last row. Each image is split vertically: the left half shows the error map (amplified ×5) of the corresponding left half of the reconstruction, while the right half displays the actual reconstructed image. A zoomed‐in region is shown to the right of each reconstruction to highlight fine details and local differences. Finally, the PSNR and SSIM of each reconstruction are indicated at the top left corner.

We first evaluate performance on cases with minimal longitudinal change. Figure 2 shows an axial $T_{2}$‐weighted example across methods and 1D acceleration rates. At low acceleration (R=3), all methods perform similarly, although LACS shows slightly more aliasing and noise. As acceleration increases, errors increase across all methods but remain consistently lower for LAPS. At R=9, AdaDiff and NERP lose structural fidelity, and LACS errors resemble the prior‐target difference map. While CAPS displays aliasing artifacts, LAPS retains a relatively clean reconstruction, showing noise‐like errors.

**FIGURE 3:**
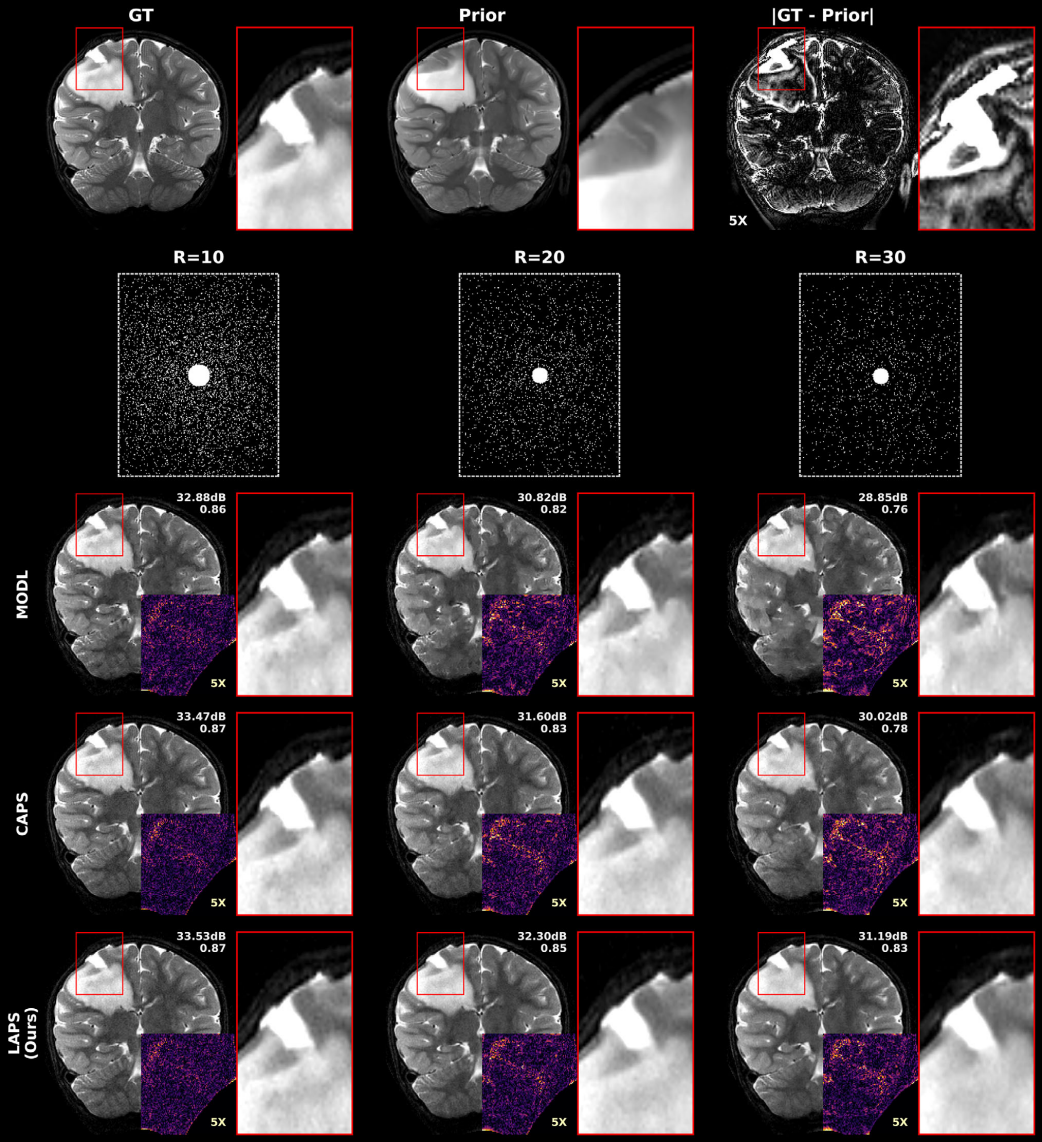
Comparison of reconstruction methods across 2D acceleration rates in a case with visible longitudinal changes. Coronal $T_{2}$‐weighted images from a pediatric brain scan with low‐grade glioma. Initial scan (prior) shows a 6cm $T_{2}$‐hyperintense mass in the right frontal and parietal lobes. In the follow‐up (target), the mass has been biopsied, with the cavity clearly visible. The first row displays (left to right): the follow‐up scan (GT), the prior, and an amplified difference highlighting changes between them. The second row shows the sampling masks used in the experiments at R=10, 20, and 30. The subsequent rows show various reconstructions, with LAPS at the bottom. The PSNR and SSIM of each method are indicated at the top right corner. A zoomed‐in region around the biopsy cavity is used to assess each method's ability to accurately reconstruct the pathology deviating from the prior. The bottom‐right quadrant is replaced with 5X amplified error.

Figure 3 shows a longitudinal case of a patient with low‐grade glioma, retrospectively evaluated at various 2D acceleration rates for the top‐performing models: LAPS, CAPS, and MODL. The prior exam shows a $T_{2}$‐hyperintense mass in the right frontal and parietal lobes. The mass was biopsied, and the resulting cavity is visible in the follow‐up scan, creating a noticeable local change.

At R=10, all methods recover the overall structure and clearly depict the cavity. At R=20, MODL exhibits undersampling artifacts and blurring, while CAPS shows emerging structural distortions. As R increases, MODL loses white/gray matter detail, but the cavity remains visible. At R=30, all methods show some degradation. LAPS demonstrates some blurring at the base of the biopsy cavity but retains lower levels of aliasing artifacts and noise than other methods.

**FIGURE 4:**
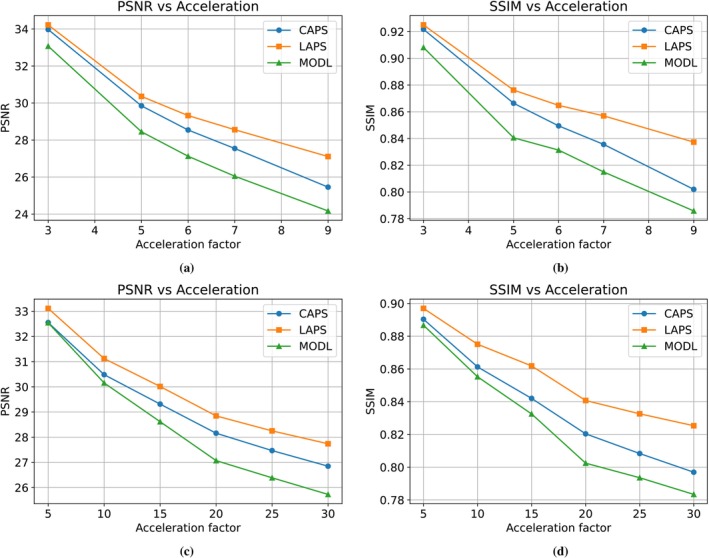
Mean global performance metrics across various 1D and 2D accelerations. For brevity, performance is only shown for LAPS with AutoInit and the top 2 performing baselines (CAPS and MODL). (a) Global PSNR versus 1D acceleration, (b) Global SSIM versus 1D acceleration, (c) Global PSNR versus 2D acceleration, (d) Global SSIM versus 2D acceleration.

Table 3 reports reconstruction metrics for 1D and 2D undersampling. LAPS consistently achieves the highest mean PSNR and SSIM across all accelerations, except for SSIM at R=3, where AdaDiff is slightly higher. At R=6, LAPS improves PSNR over CAPS by 0.8dB, increasing to 1.65dB at R=9. Similar trends are observed in 2D undersampling. Figure 4 shows PSNR and SSIM versus R per method, where LAPS at R=30 (2D) matches or exceeds CAPS at about R=23. In the 1D case, LAPS at R=9 is quantitatively comparable to CAPS at R=7.

**TABLE 3:** Comparison of PSNR and SSIM (Mean ± SD) for different methods on the test set under 1D acceleration (top block) and 2D acceleration (bottom block) at various rates. Best‐performing results are highlighted in **bold**. The rows labeled *Target–Prior* reports the metric values computed between the registered target and the prior scans, serving as a baseline for their intrinsic similarity.

		PSNR			SSIM		
	Method	R=3	R=6	R=9	R=3	R=6	R=9
*1D*	LAPS (Ours)	**34.22** ± **3.65**	**29.32** ± **3.17**	**27.11** ± **2.94**	0.925±0.049	**0.865** ± **0.069**	**0.837** ± **0.076**
	Target‐Prior	19.93±1.91	19.93±1.91	19.93±1.91	0.677±0.097	0.677±0.097	0.677±0.097
	AdaDiff	33.91±3.14	26.93±2.44	23.26±2.12	**0.931** ± **0.045**	0.832±0.069	0.761±0.079
	CAPS	33.97±3.66	28.54±3.10	25.46±2.80	0.922±0.050	0.849±0.072	0.802±0.081
	CG‐SENSE	30.00±3.57	24.63±2.19	22.30±1.91	0.859±0.075	0.778±0.077	0.731±0.079
	LACS	32.61±3.08	26.40±2.28	23.79±2.04	0.920±0.048	0.829±0.064	0.784±0.069
	MODL	33.07±4.16	27.13±3.43	24.17±3.05	0.908±0.061	0.831±0.077	0.786±0.083
	NERP	31.09±2.71	26.31±2.54	24.36±2.40	0.904±0.055	0.822±0.076	0.792±0.080

		R = 10	R = 20	R = 30	R = 10	R = 20	R = 30
*2D*	LAPS (Ours)	**31.12** ± **2.93**	**28.85** ± **3.44**	**27.74** ± **3.20**	**0.875** ± **0.042**	**0.841** ± **0.054**	**0.825** ± **0.056**
	Target‐Prior	20.11±2.06	20.11±2.06	20.11±2.06	0.688±0.106	0.688±0.106	0.688±0.106
	AdaDiff	29.70±3.27	25.91±3.13	23.84±2.67	0.862±0.051	0.790±0.066	0.746±0.069
	CAPS	30.48±2.92	28.16±3.38	26.85±3.13	0.861±0.045	0.820±0.057	0.797±0.059
	CG‐SENSE	28.01±2.83	25.37±2.71	24.06±2.25	0.803±0.063	0.759±0.068	0.732±0.069
	LACS	29.58±2.65	26.62±2.53	25.22±2.14	0.861±0.045	0.810±0.054	0.784±0.056
	MODL	30.15±3.98	27.07±4.32	25.73±3.91	0.855±0.058	0.802±0.074	0.783±0.073
	NERP	28.54±2.80	26.18±2.97	25.20±2.65	0.854±0.051	0.803±0.063	0.783±0.062

**FIGURE 5:**
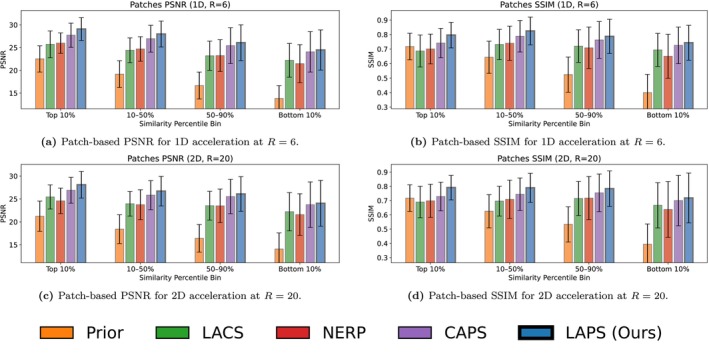
Patch‐based metric analysis. Each slice in the test set was divided into 32×32 patches with 50% overlap, and the similarity between corresponding patches in the target and prior scans was computed using cosine similarity. To evaluate performance as a function of prior similarity, we aggregated all patches across the test set and grouped them into bins based on similarity percentiles. In each subplot, the x‐axis represents a similarity percentile bin: the leftmost bin includes the 10% most similar patches, followed by patches in the 10–50% range (still similar but less so), then the 50–90% range, and finally, the rightmost bin includes the 10% most dissimilar patches. The bar labeled Prior shows the metric computed between the target and prior patches within each range. (a) Patch‐based PSNR for 1D acceleration at R=6. (b) Patch‐based SSIM for 1D acceleration at R=6. (c) Patch‐based PSNR for 2D acceleration at R=20. (d) Patch‐based SSIM for 2D acceleration at R=20. (e) Legend for patch‐based metrics.

For patch‐based analysis, Figure 5 compares all longitudinal reconstruction methods and the top‐performing non‐longitudinal baseline (CAPS) at various similarity percentiles for R=6 (1D) and R=20 (2D). We also include a baseline computed between corresponding patches from the ground truth and prior scans. In the most similar patches, LAPS exceeds CAPS by 1–2 dB in PSNR and 10% in SSIM, with even larger gains over other longitudinal methods. As similarity decreases, the gap narrows, and in the most dissimilar patches, LAPS and CAPS perform comparably.

### 4.2 Performance With Varying Levels of Change

**FIGURE 6:**
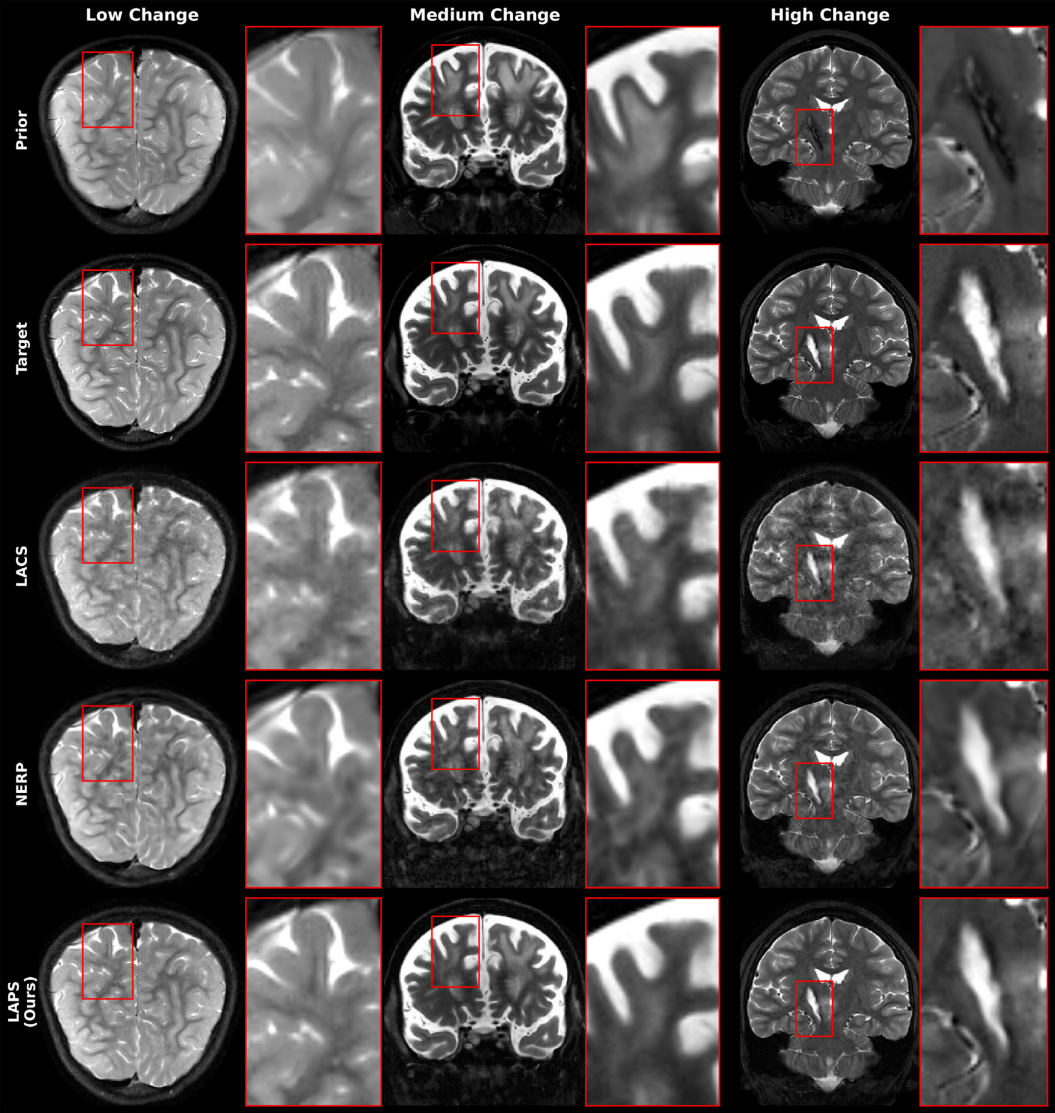
Comparison of different longitudinal reconstruction algorithms across varying degrees of change between scans, for a 2D undersampling case with R=20. The first row shows the prior scan, the second row shows the new scan (target), and the subsequent rows present reconstructions from different methods, with our proposed method (LAPS) shown in the last row. Left column: Axial $T_{2}$‐weighted slice from the same patient shown in Figure 3, now displaying a region with minimal or no change between scans. Middle column: Coronal $T_{2}$‐weighted images of a patient with a brainstem tumor (not shown). The displayed slice shows $T_{2}$‐hyperintense white matter changes related to treatment, along with white matter swelling that decreases on the follow‐up MRI performed 6 days later. Right column: Coronal $T_{2}$‐weighted images of a brain with an involuted right basal ganglia hematoma, secondary to a ruptured arteriovenous malformation. The initial exam reveals a $T_{2}$‐hypointense contracted cavity corresponding to old blood products. The follow‐up exam, performed 4 months later, shows fluid accumulation within the cavity and $T_{2}$‐hyperintense edema in the surrounding brain parenchyma due to interval radiosurgery treatment. The automatically selected projection times $t_{p}$, estimated by AutoInit, were 280, 332, and 453 for the left, middle, and right columns, respectively, reflecting increasing levels of change across the three cases.

Figure 6 compares longitudinal reconstruction methods across three subjects with increasing levels of change between scan timepoints, as assessed by a radiologist (see Table 2b). All reconstructions use 2D undersampling at R=20. LACS shows undersampling artifacts in all cases, worsening with greater anatomical change. NERP introduces structural and contrast‐related artifacts throughout. In contrast, LAPS maintains consistent reconstruction quality across all levels of change. Additional high‐resolution reconstruction examples with different changes and contrast are provided in Supplementary Figures S6, S7, and S8.

### 4.3 Performance With Misregistration

**FIGURE 7:**
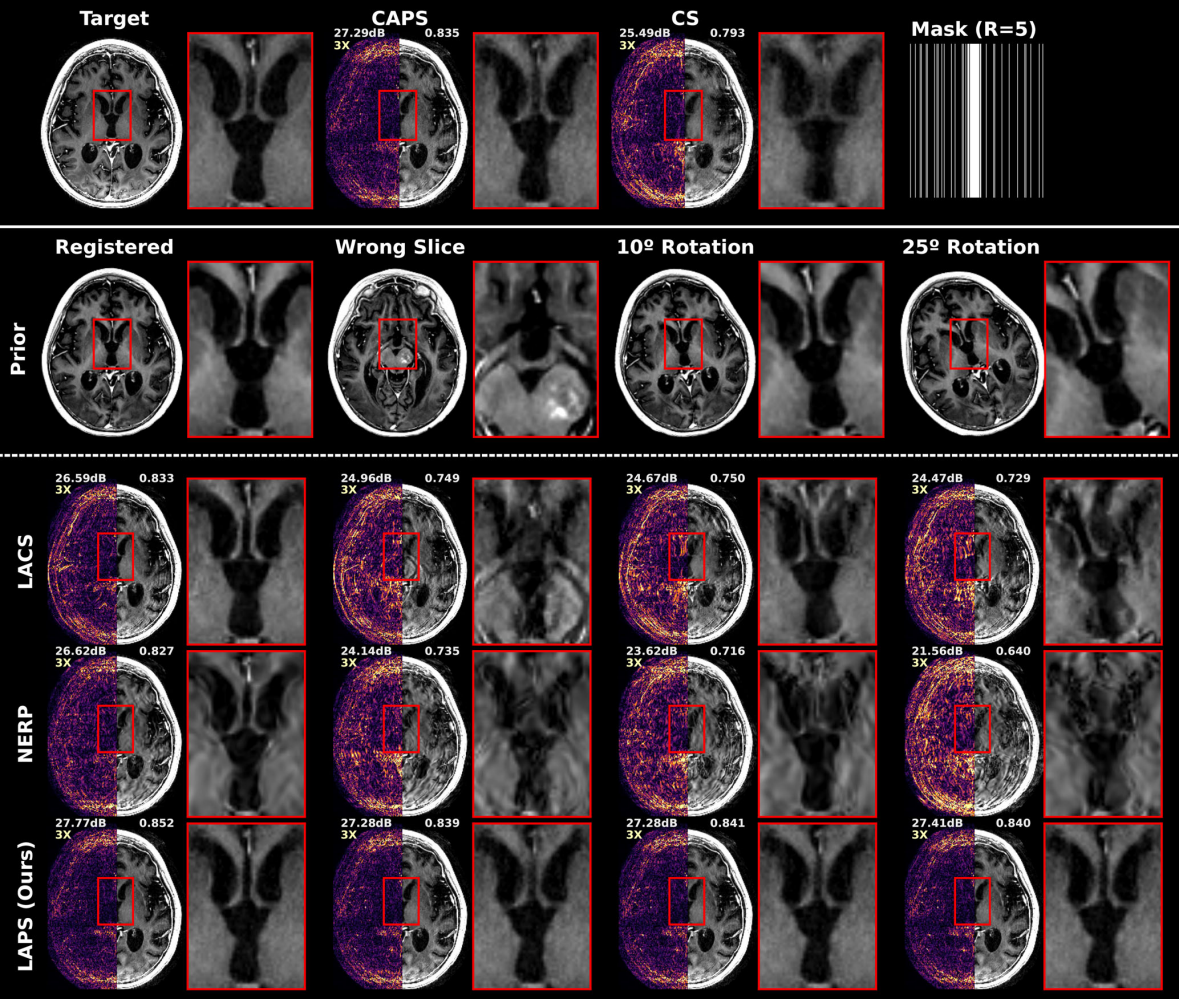
Reconstruction results of longitudinal methods under various misregistration scenarios, for Axial $T_{1}$‐BRAVO acquisition with R=5 1D undersampling. Top row shows the target scan, two non‐longitudinal baselines (CAPS and L1‐Wavelet CS, which is equivalent to LACS without regularization on the prior image), and the undersampling mask. Below, each column exhibits a different prior registration scenario atop the corresponding longitudinal reconstructions. First is perfect registration, followed by the selection of a prior slice 10mm off from the target slice, finally with mis‐alignment of the correct prior slice by $10^{\circ }$ and $25^{\circ }$ in‐plane rotation offsets, respectively. PSNR and SSIM are shown in the top‐left and top‐right of each image, respectively. The automatically selected projection times $t_{p}$, estimated by AutoInit for LAPS were 300, 450, 560, and 600 (left to right), reflecting decreased levels of reliance on the prior with increased mis‐registration.

Previous results assumed good alignment between the prior and follow‐up scans. To evaluate robustness to misregistration, Figure 7 shows reconstructions of a $T_{1}$‐weighted scan at R=5 (1D) with various misregistrations, including in‐plane rotation and slice mismatch.

Without misregistration, all longitudinal methods perform comparably to non‐longitudinal baselines. Under rotation, LACS and NERP exhibit the most severe error, with visible residuals from the misaligned prior. Slice mismatch causes structural interference in both methods, most notably in LACS, which appears as a superposition of the incorrect prior and the follow‐up scan. NERP degrades noticeably with increasing misregistration, showing incoherent artifacts. Quantitatively, PSNR drops by 1.5dB or more for LACS and NERP, with NERP decreasing as much as 5dB with the high rotation case. In contrast, LAPS drops by only 0.5dB, maintaining performance comparable to CAPS even under misregistration. Notably, the $t_{p}$ chosen by AutoInit adapts to the level of misregistration, increasing with misalignment severity (see figure caption for $t_{p}$ values).

## 5 Discussion

### 5.1 Effectiveness of Diffusion‐Based Priors in Longitudinal Reconstruction

One of the key factors contributing to the increased performance of LAPS is the use of a powerful LDM trained on large‐scale, unpaired data. This enables LAPS to learn a flexible and expressive image prior, in contrast to LACS, which relies on a handcrafted sparsity prior, and NERP, which uses an INR trained on the prior scan without learning from a broader data distribution. As shown in Table 3, LAPS consistently outperforms all other methods across 1D and 2D accelerations, maintaining high image quality even at high R. The advantage of the LDM prior is further supported by CAPS, which also utilizes an LDM and shows strong performance compared to the other non‐longitudinal methods. Additionally, LDM training was noticeably faster than image‐space models like AdaDiff, taking roughly half the time to converge.

#### 5.1.1 Hot‐Starting a Diffusion Process

Several works have explored non‐pure noise initializations for diffusion models. In image‐editing methods [28, 29], noise is added to a reference image, or the diffusion trajectory is inverted to enable semantic or text‐guided edits. The initial timestep is not optimized, but instead chosen by the user or set by the inversion procedure. These approaches lack a measurement model and do not enforce DC, since their aim is to modify an existing image rather than reconstruct one from physical measurements.

In MRI reconstruction, “cold diffusion” strategies [49, 50] initialize sampling from the zero‐filled reconstruction and propagate the diffusion process using k‐space undersampling instead of Gaussian noise. Other works similarly begin from a zero‐filled [51, 52] or low‐frequency reconstruction to speed‐up convergence. These initializations can reduce sampling time but are fixed by the forward model and do not incorporate information beyond the acquired k‐space. In contrast, our approach leverages a subject‐specific prior scan as additional information and selects the starting point based on an estimate of similarity between acquisitions.

### 5.2 Comparison With Baseline Longitudinal Methods

To assess how longitudinal methods handle scan differences, we evaluated performance across varying anatomical change and misregistration. As shown in the patch‐based analysis (Figure 5), LAPS more effectively leverages regions with high prior similarity. This is also illustrated in Figure 2, where the prior and follow‐up scans differ minimally. In such cases, LAPS selects a smaller $t_{p}$ (around 200, compared to the typical range of 250‐500 observed in the test set), allowing the diffusion process, guided by DC steps, to stay close to the prior while incorporating subtle changes from the measurements.

Importantly, even in regions where the prior offers limited information, LAPS performs on par with or better than CAPS, indicating it does not over‐rely on the prior. Instead, it uses high‐similarity regions to better condition the global reconstruction, benefiting dissimilar areas as well. No performance degradation is observed in low‐similarity patches compared to CAPS, suggesting LAPS effectively balances the LDM and DC, even when the prior is uninformative.

In contrast, LACS's wavelet sparsity prior becomes insufficient at higher acceleration rates, where the inverse problem is more ill‐posed. Furthermore, its prior‐weighting scheme depends on intermediate reconstructions, which may be highly degraded under heavy undersampling, limiting its ability to incorporate useful prior information.

NERP performs well when prior and current scans are similar but degrades under larger anatomical changes or severe undersampling (Figure 6). In such cases, the INR, which separates use of the prior scan and DC into two different optimization stages, does not have sufficient constraints and often results in overly smoothed or anatomically implausible reconstructions.

Notably, Figure 5 shows that CAPS performance declines with lower patch similarity, despite it not being a longitudinal method. This is due to the clinical data containing spatially varying residual noise, causing low cosine similarity in regions with lower SNR rather than true anatomical differences. Consequently, dissimilar patches often have higher noise, reducing metric values for all methods.

### 5.3 Robustness to Anatomical Change and Misregistration

A key requirement for longitudinal reconstruction is preserving meaningful differences between scans. As demonstrated in Figures 3 and 6, LAPS adapts to subtle and large‐scale anatomical differences effectively. Its ability to gradually diverge from the prior initialization during reconstruction allows it to capture new pathological structure without forcing agreement with the prior scan.

Additionally, though registering scan timepoints is typically straightforward, it remains important to be robust to misregistration, which can still occur especially when registering to a low‐resolution navigator or initial reconstruction. As shown in Figure 7, LAPS facilitates such flexibility via AutoInit, selecting higher $t_{p}$ with increased prior mis‐alignment. Supplementary Figure S2c additionally shows an ablation on the starting timestep, where notably using the $t_{p}$ from AutoInit outperforms any fixed choice of $t_{p}$, highlighting the specific utility of adaptive initialization. In contrast, pixel‐domain methods like LACS and NERP exhibit artifacts from misaligned priors. We further attribute LAPS's robustness to mis‐registration to (1) the use of latent representations that are less sensitive to spatial misalignment and (2) the iterative nature of the diffusion process, which can correct for spatial mismatches by gradually refining the output to match acquired measurements.

### 5.4 Limitations and Future Work

LAPS showcases a feasible approach for improving reconstruction quality with prior scan information, but remains computationally complex. LDM inference requires iterative sampling and repeated DC steps, which is slower than unrolled networks like MODL that can reconstruct each slice within 1‐2 s. However, we note that the LAPS reconstruction time is comparable with AdaDiff and NERP at about 2‐3 min per slice, as all three require iterative multi‐stage fine‐tuning at inference. Future work could explore efficient approximations of the DC update, potentially using learned or linearized operators to reduce runtime.

Additionally, we observed that LAPS, like all methods, experiences degradation at high accelerations, particularly R≈30 in 2D. This performance cap is partially limited by the size and variable quality of SLAM training data, but also from our choice to validate on retrospective clinical cases which undersample k‐space suboptimally. To push for higher acceleration, one potential direction is to design sampling patterns tailored for longitudinal imaging. Although previously proposed adaptive k‐space sampling methods like those in LACS [26] may not yet be practical clinically, an offline prior‐informed strategy for generating k‐space sampling patterns could further improve LAPS at high accelerations.

Since SLAM is a clinical dataset, scans are acquired with mild undersampling and without averaging, so the reference images contain non‐negligible noise. Consequently, the achievable quantitative metrics are fundamentally limited by this noise level. This differs from some reconstruction studies that use fully sampled, often multi‐averaged images that provide clean, high‐SNR ground‐truth labels, and therefore yield higher metric values. Importantly, the relative differences between methods remain meaningful and consistent, which is the primary value of these quantitative assessments. Developing training strategies that better account for noisy targets [53, 54] is an important direction for future work as the SLAM data set continues to expand.

Though LAPS provides a practical workaround for the absence of paired longitudinal training data, a model that explicitly learns the longitudinal conditional distribution could, in principle, reduce reliance on heuristic components, simplify the method, and potentially outperform our current approach. Nevertheless, our initialization strategy is easy to implement into existing reconstruction strategies, and thus could easily be extended to work synergistically with contrast‐to‐contrast conditioning or other reconstruction frameworks. Such synergy could further refine reconstruction quality, and could even be benchmarked with the multi‐contrast SLAM data set, but likely requires more intensive compute and data given the addition of another representation axis.

## 6 Conclusion

The longitudinal application of MRI is vast and is only growing in utility, especially in neuroimaging, both for large‐scale research studies as well as numerous clinical settings tracking disease or structural progression [55]. Consecutive scans of the same subject often contain rich mutual information that, if properly leveraged, can improve reconstruction quality and reduce acquisition time. However, most state‐of‐the‐art reconstruction methods require large, paired datasets for training. This requirement is difficult to satisfy in longitudinal settings, particularly when raw k‐space data are needed.

To address this, we proposed LAPS, an LDM‐based framework that operates without requiring paired training data. LAPS learns a general image prior from unpaired data and incorporates subject‐specific prior scans during inference via a latent‐space projection, enabling flexible use of scan priors while preserving clinically meaningful changes. To support evaluation, we also introduce SLAM, a multi‐contrast, multi‐session clinical data set with both healthy and pathological cases. SLAM contains raw k‐space from follow‐up scans, a large portion of which have corresponding prior DICOM scans. The paired subset of SLAM was used here primarily for inference but has potential for supervised training as it grows.

Our experiments show that LAPS consistently outperforms both longitudinal and non‐longitudinal baselines across acceleration rates, anatomical changes, and misregistration scenarios. It effectively leverages prior scans when informative and remains robust when they are not. By combining a general LDM prior with subject‐specific guidance at inference, LAPS offers a flexible and principled approach to longitudinal reconstruction.

## Funding

This work was supported by the National Institutes of Health (Grant No. R01EB019437, R01MH116173).

## Supporting information




**Data S1: Figure S1.** Illustration of optimal $t_{p}$ selection versus the prior‐target difference as measured by maximization of $\log p({\text{z}}^{⋆}|{\text{z}}^{P})$. Examples of $t_{p}$ objective function shown for image pairs at two different levels of similarity.
**Figure S2.** Calibration development of AutoInit for automatic $t_{p}$ selection. Relationships between the theoretical, approximate, and empirically optimal $t_{p}$'s are linearly regressed, showing strong linear correlation. Linear model which produces the affine parameters used in Algorithm 3 is shown. The calibrated AutoInit algorithm is validated against a parameter gridsearch on an unseen test set, outperforming PSNR for all fixed choices of $t_{p}$.
**Figure S3.** Comparison of latent posterior sampling inference methods via image quality, quantitative metrics, and reconstruction time. Reconstructions with the same trained network for various inference methods compared: the original PLDS algorithm [42], PLDS initialized at $t_{p}=200$ with a CG‐SENSE recon, our CAPS method, and our proposed LAPS method. LAPS also shown with networks at 4× and 8× down‐sampling, highlighting the speed/performance tradeoff for latent compression.
**Figure S4.** Comparative examples of retrospective undersampling masks. (a) shows 1D undersampling of data acquired with a 1D mask at various rates, as well as a truly fully sampled mask. (b) shows analog 2D cases.
**Figure S5.** Performance of reconstruction metrics for various $n_{\text{opt}}$, with $t_{p}$ chosen via AutoInit, for $n_{\text{step}}=100$ DDIM steps. $n_{\text{opt}}=1$ equates to sampling with PLDS [42]; for both CAPS and LAPS, $n_{\text{opt}}=10$ maximizes PSNR and SSIM in the reconstruction test set. LAPS consistently boosts performance compared to CAPS for each value of $n_{\text{opt}}$, showing the utility of the prior scan.
**Figure S6.** Additional reconstruction example for all methods at R=7 with 1D undersampling, shown as an axial view for the large change coronal example in Figure 6.
**Figure S7.** Additional reconstruction example for all methods at R=20 with 2D undersampling for a $T_{2}$‐FLAIR contrast.
**Figure S8.** Additional reconstruction example for all methods at R=7 with 1D undersampling, for an example where the prior contrast slightly differs from the new scan contrast. **Table S1.** Specific breakdown of scans in SLAM data set by protocol and scan acquisition type, categorized by the total number of scans with raw k‐space data (All) and the subset that are paired to a prior scan (Pair). Composition further described by split into train and test data sets.

## Data Availability

The data that support the findings of this study are openly available in Stanford Digital Repository at https://doi.org/10.25740/rq296rb2765.

## Appendix A Derivation for Projection Timepoint $t_{p}$ in the Gaussian Case

We compute the optimal projection point $t_{p}$ for the case ${\text{z}}_{0}∼𝒩(0,\text{I})$, as the typical training objective of VAEs minimizes divergence between the learned p(z) and a normal Gaussian [36]. Beginning with Equation (12):

(A1)
$$\begin{array}{ll} t_{p} & =\underset{t}{\arg\;\max}p_{0}({\text{z}}_{0}={\text{z}}^{⋆}|{\text{z}}_{t}={\text{z}}_{t}^{P}) \end{array}$$

(A2)
$$\begin{array}{ll} & =\underset{t}{\arg\;\max}\frac{p_{0}({\text{z}}_{0}={\text{z}}^{⋆}|{\text{z}}_{t}={\text{z}}_{t}^{P})}{p_{0}({\text{z}}_{0}={\text{z}}^{⋆})} \end{array}$$

(A3)
$$\begin{array}{ll} & =\underset{t}{\arg\;\max}\frac{p_{t}({\text{z}}_{t}={\text{z}}_{t}^{P}|{\text{z}}_{0}={\text{z}}^{⋆})}{p_{t}({\text{z}}_{t}={\text{z}}_{t}^{P})} \end{array}$$

(A4)
$$\begin{array}{ll} & =\underset{t}{\arg\;\max}\frac{p_{t}({\text{z}}_{t}=\sqrt{{\bar{\alpha }}_{t}}{\text{z}}^{P}+\sqrt{1-{\bar{\alpha }}_{t}}\epsilon |{\text{z}}_{0}={\text{z}}^{⋆})}{p_{t}({\text{z}}_{t}=\sqrt{{\bar{\alpha }}_{t}}{\text{z}}^{P}+\sqrt{1-{\bar{\alpha }}_{t}}\epsilon )} \end{array}$$

(A5)
$$\begin{array}{ll} & =\underset{t}{\arg\;\max}\frac{𝒩(\sqrt{{\bar{\alpha }}_{t}}{\text{z}}^{P};\sqrt{{\bar{\alpha }}_{t}}{\text{z}}^{⋆},2(1-{\bar{\alpha }}_{t}))}{𝒩(\sqrt{{\bar{\alpha }}_{t}}{\text{z}}^{P};0,2-{\bar{\alpha }}_{t})} \end{array}$$

(A6)
$$\begin{array}{ll} & =\underset{t}{\arg\;\max}\frac{\sqrt{2-{\bar{\alpha }}_{t}}}{\sqrt{2(1-{\bar{\alpha }}_{t})}}\cdot \frac{\exp\left\{-\frac{{\bar{\alpha }}_{t}}{4(1-{\bar{\alpha }}_{t})}\|{\text{z}}^{⋆}-{\text{z}}^{P}{\|}_{2}^{2}\right\}}{\exp\left\{-\frac{{\bar{\alpha }}_{t}}{2(2-{\bar{\alpha }}_{t})}\|{\text{z}}^{P}{\|}_{2}^{2}\right\}} \end{array}$$

(A7)
$$\begin{array}{ll} & =\underset{t}{\arg\;\max}\gamma_{t}+\frac{{\bar{\alpha }}_{t}}{2}\left[\frac{\|{\text{z}}^{P}{\|}_{2}^{2}}{2-{\bar{\alpha }}_{t}}-\frac{\|{\text{z}}^{⋆}-{\text{z}}^{P}{\|}_{2}^{2}}{2(1-{\bar{\alpha }}_{t})}\right] \end{array}$$

Where,

(A8)
$$\gamma_{t}=\frac{1}{2}\log\left(\frac{2-{\bar{\alpha }}_{t}}{2(1-{\bar{\alpha }}_{t})}\right)$$

The derivation steps follow as Step (A2) divides by a term independent of t; Step (A3) applies Bayes' rule; Step (A4) substitutes the definition of ${\text{z}}_{t}$ from Equation (11); and Step (A5) uses the forward diffusion process, with ϵ∼𝒩(0,I) independent of ${\text{z}}_{0}∼𝒩(0,\text{I})$.
