# Supplementary material for: Accelerating MRI With Longitudinally‐Informed Latent Posterior Sampling
Source: Magn Reson Med. 2026 Feb 22;95(6):3445–61. doi: 10.1002/mrm.70257 (PMC13049276; doi:10.1002/mrm.70257)
Supplement: Supplementary file 1 — Data S1: Figure S1. Illustration of optimal tp selection versus the prior‐target difference as measured by maximization of logp(z⋆|zP). Examples of tp objective function shown for image pairs at two different levels of similarity. Figure S2. Calibration development of AutoInit for automatic tp selection. Relationships between the theoretical, approximate, and empirically optimal tp's are linearly regressed, showing strong linear correlation. Linear model which produces the affine parameters used in Algorithm 3 is shown. The calibrated AutoInit algorithm is validated against a parameter gridsearch on an unseen test set, outperforming PSNR for all fixed choices of tp. Figure S3. Comparison of latent posterior sampling inference methods via image quality, quantitative metrics, and reconstruction time. Reconstructions with the same trained network for various inference methods compared: the original PLDS algorithm [42], PLDS initialized at tp=200 with a CG‐SENSE recon, our CAPS method, and our proposed LAPS method. LAPS also shown with networks at 4× and 8× down‐sampling, highlighting the speed/performance tradeoff for latent compression. Figure S4. Comparative examples of retrospective undersampling masks. (a) shows 1D undersampling of data acquired with a 1D mask at various rates, as well as a truly fully sampled mask. (b) shows analog 2D cases. Figure S5. Performance of reconstruction metrics for various nopt, with tp chosen via AutoInit, for nstep=100 DDIM steps. nopt=1 equates to sampling with PLDS [42]; for both CAPS and LAPS, nopt=10 maximizes PSNR and SSIM in the reconstruction test set. LAPS consistently boosts performance compared to CAPS for each value of nopt, showing the utility of the prior scan. Figure S6. Additional reconstruction example for all methods at R=7 with 1D undersampling, shown as an axial view for the large change coronal example in Figure 6. Figure S7. Additional reconstruction example for all methods at R=20 with 2D undersampling for a T2‐ [file MRM-95-3445-s001.pdf]

S1 | SUPPLEMENTARY FIGURES

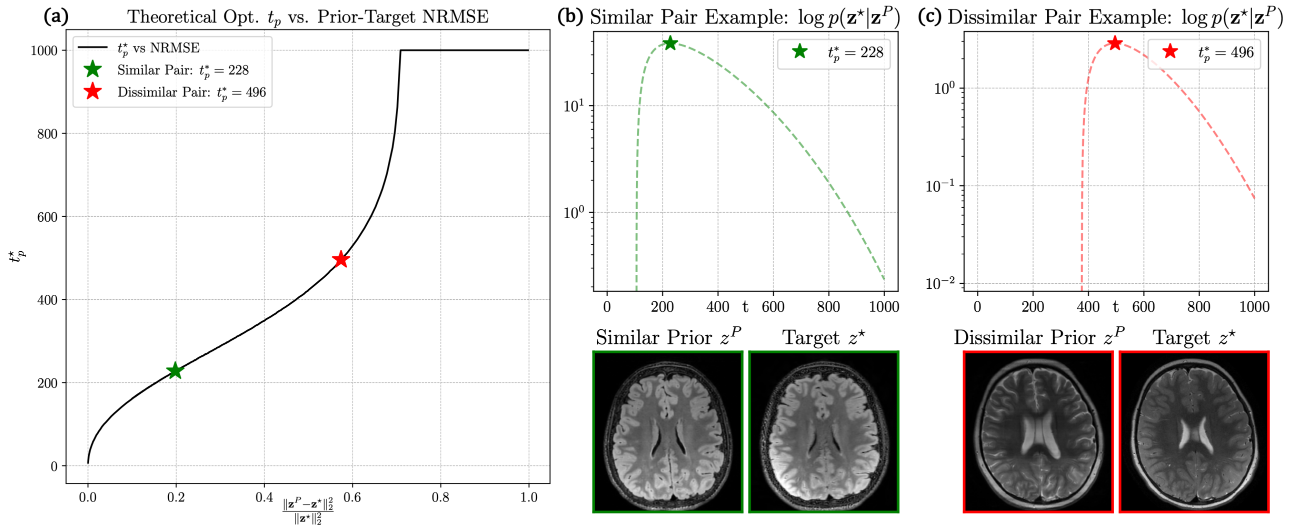

FIGURE S1 Illustration of theoretically derived t_p selection vs. prior-target difference. (a) shows the theoretically optimal t_p from Eq. (13) as a function of $\mathtt{NRMSE}(\mathbf{z}^{\mathrm{P}}, \mathbf{z}^\star) = \|\mathbf{z}^{\mathrm{P}} - \mathbf{z}^\star\|_2^2 / \|\mathbf{z}^\star\|_2^2$ when $\mathbf{z}^\star \sim \mathcal{N}(0, \mathbf{I})$ is sampled randomly, and the prior differs by a random noise vector: $\mathbf{z}^{\mathrm{P}} = \mathbf{z}^\star + \mathbf{n}$ for $\mathbf{n} \sim \mathcal{N}(0, \sigma^2\mathbf{I})$. This shows theoretical performance at different levels of additive noise σ and thus different prior-target NRMSE. (b) and (c) show examples of the objective in Eq. (13) which proxies $\log p_0(\mathbf{z}^\star \mid \mathbf{z}_t = \mathbf{z}_t^{\mathrm{P}})$ for real image pairs, with the respective curve maxima as the selected $t_p^\star$'s. (b) shows a case with high scan similarity (low NRMSE), and (c) a case with more image dis-similarity (high NRMSE), seen by visible difference in contrast and morphology in the central ventricles. The optimal t_p for our learned latent space is some intermediate timestep for both cases, with higher image dis-similarity favoring higher $t_p^\star$.

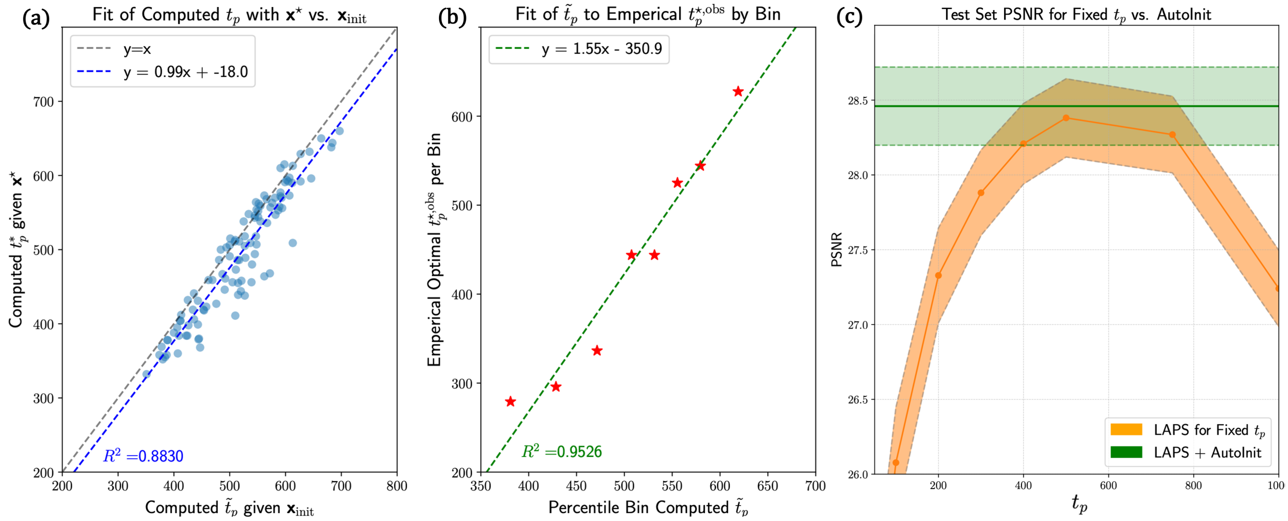

FIGURE S2 Development of `AutoInit` for Automatic t_p Selection. To empirically validate and calibrate `AutoInit`, we run LAPS at several fixed values of t_p over multiple 1D & 2D undersampling rates in the calibration set $\mathfrak{D}_v$, which is comprised of 100 registered and un-registered prior-target pairs from 10 subjects. Per pair, we also compute $\tilde{t}_p$ via Eq. (15) using a fast CAPS recon for $\mathbf{x}_{\text{init}}$. We validate that Eq. (15) is a good approximation for Eq. (13) in (a), where the t_p computed given the ground truth $\mathbf{x}^\star$ and prior $\mathbf{x}^{\mathrm{P}}$ correlates to our computed $\tilde{t}_p$ given $\mathbf{x}_{\text{init}}$ and $\mathbf{x}^{\mathrm{P}}$ with $R^2 = 0.883$ and trendline slope of nearly 1. We then split $\mathfrak{D}_v$ into 8 bins over percentiles of the computed $\tilde{t}_p$, and found an empirical optimum $t_p^{\star,\text{obs}}$ for each bin as the fixed t_p which maximized the average reconstruction PSNR and SSIM. We then fit a linear model between $\tilde{t}_p$ and $t_p^{\star,\text{obs}}$ over bins in (b), finding $v_{\mathrm{p}} = 1.55, w_{\mathrm{p}} = -350$ from the regression slope and intercept as the `AutoInit` calibration parameters. To validate this heuristic, (c) compares performance of this calibrated `AutoInit` (green) on a held-out test set against fixed choices of t_p (orange), shown as mean PSNR $\pm$ standard error. For fixed t_p, as t_p increases, initially PSNR increases, but with too high of t_p, performance decreases. Note that $t_p = 1000 = T$ is equivalent to unconditional inference, as the initialization is pure noise. `AutoInit` outperforms all choices of fixed t_p, demonstrating the flexibility of this prior initialization.

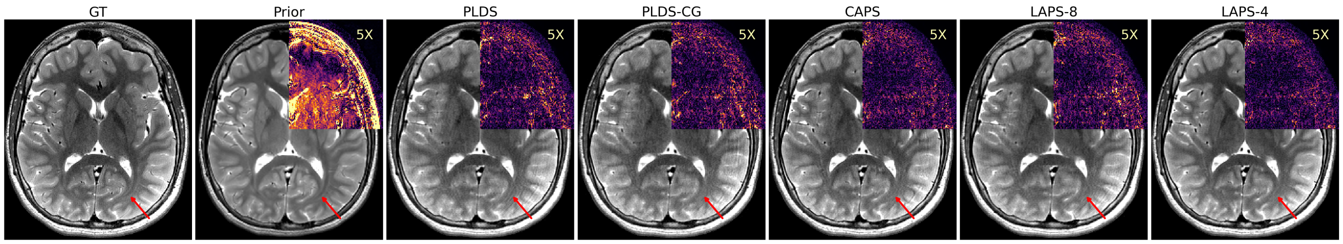

(a) Example Reconstruction with various latent posterior sampling methods.

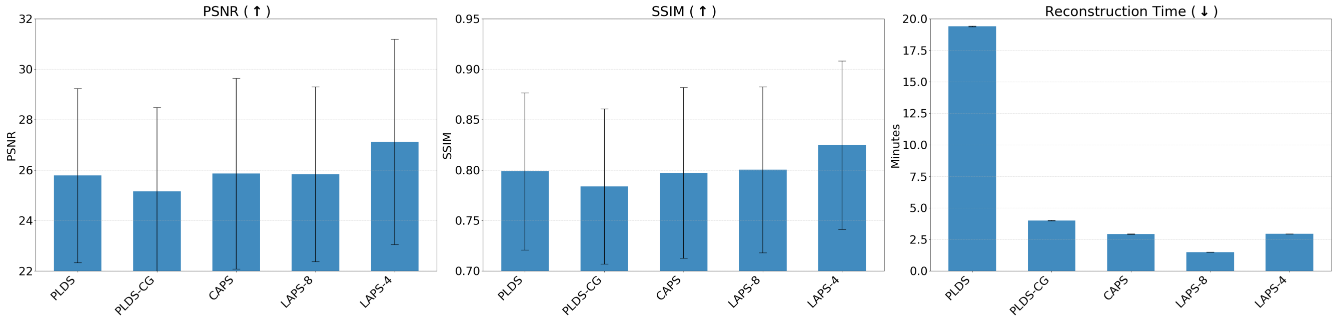

(b) Cumulative Metrics over a subset of 40 slices in the test set, with a mix of R=25 acceleration with 2D undersampling, and R=7 acceleration with 1D undersampling.

FIGURE S3 Comparison of Latent Posterior Sampling Inference Methods. All methods use the same trained networks with VAE downsampling of $K = 4$, with the exception of LAPS-8, which uses equivalent networks with further downsampling to $K = 8$. (a) shows a typical reconstruction example with the upper-right quartile of the image replaced with 5x error with respect to the ground truth (GT). From left to right: PLDS[42], an extension of LDPS which uses a DDPM sampler for one latent gradient step per DC update, requires substantially longer reconstruction times, and undersampling artifacts are still visible. PLDS-CG is an initialization of PLDS at $t_p = 200$ with a CG-SENSE recon, decreasing reconstruction time 5x as shown in (b), but with a minor performance hit. CAPS, our proposed latent sampling method without a prior scan, is also initialized at $t_p = 200$ with the CG-SENSE recon, but instead uses a DDIM sampler with repeated DC for $n_{\text{opt}} = 10$, speeding up reconstruction further with equivalent performance to PLDS. Finally, LAPS-8 and LAPS-4 show our method at different down-sampling levels, highlighting the speed/performance tradeoff with latent compression.

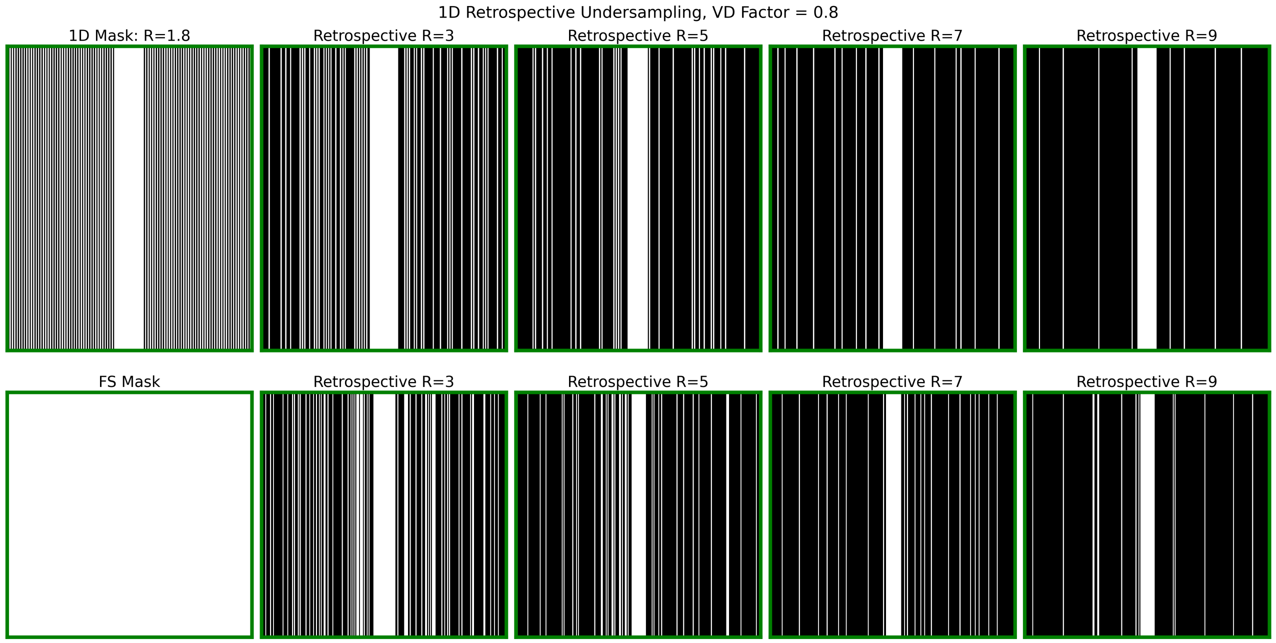

(a) Example Retrospective Undersampling with 1D mask from 1D undersampled scan compared to fully sampled scan.

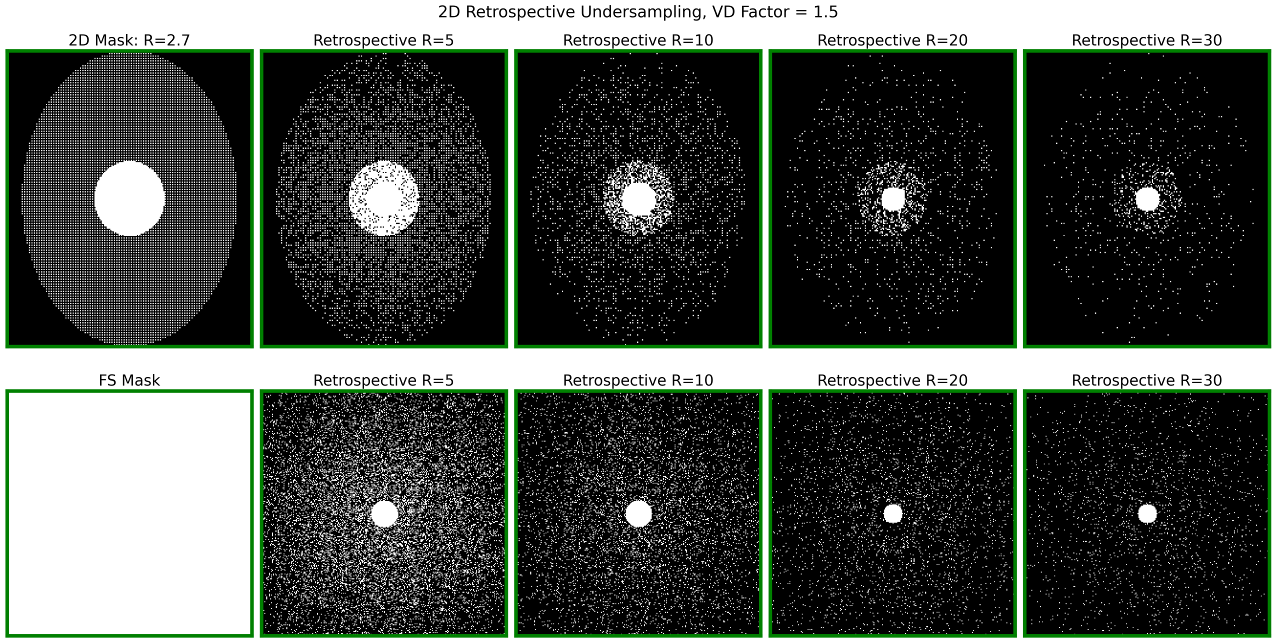

(b) Example Retrospective Undersampling with 2D mask from 2D undersampled scan compared to fully sampled scan.

FIGURE S4 Comparison of retrospective undersampling masks. (a) shows 1D undersampling of data acquired with a 1D mask (top) at various rates, as well as a truly fully sampled mask (bottom). (b) shows the analog 2D cases.

Protocol	Total	Train		Test
	**All**	**All**	Pair	**Pair**
**Brain Tumor**	110	94	36	14
**Brain & Pituitary**	29	29	14	-
**Glioma/Radiation**	23	23	-	-
**Brain & Orbits**	19	19	12	-
**Stroke**	9	9	-	-
**Ped Brain**	13	8	8	5
**ROSA**	7	7	6	-
**Brain Vascular**	8	6	3	2
**Seizures**	4	4	2	-
**Epilepsy**	3	3	-	-
**Other**	7	7	-	-

(a) Distribution of scans by protocol.

Scan Type	Total	Train		Test
	**All**	**All**	Pair	**Pair**
T_2 **(2D)**	83	72	40	11
T_2 **FLAIR (3D)**	40	37	17	3
T_1 **BRAVO (3D)**	24	23	7	1
T_1 **BRAVO+GAD (3D)**	23	19	9	4
T_2 **(3D)**	21	18	4	3
T_1 **FSE (3D)**	15	15	-	-
T_1 **CUBE (3D)**	11	10	2	1
T_2 **FLAIR (2D)**	7	7	2	-
T_1 **SPGR (3D)**	5	5	-	-
T_1 **FLAIR (2D)**	3	3	-	-
**Other**	-	-	-	-

(b) SLAM scans by scan type.

TABLE S1 Specific breakdown of scans in SLAM dataset, categorized by the total number of scans with raw k-space data (All), which can be used for training the LDM, and the subset that are paired, i.e., include a matching prior scan (Pair), which can be used for validating our method. SLAM was split by subject into train and test datasets, with test set scans filtered only to paired examples required for evaluation.

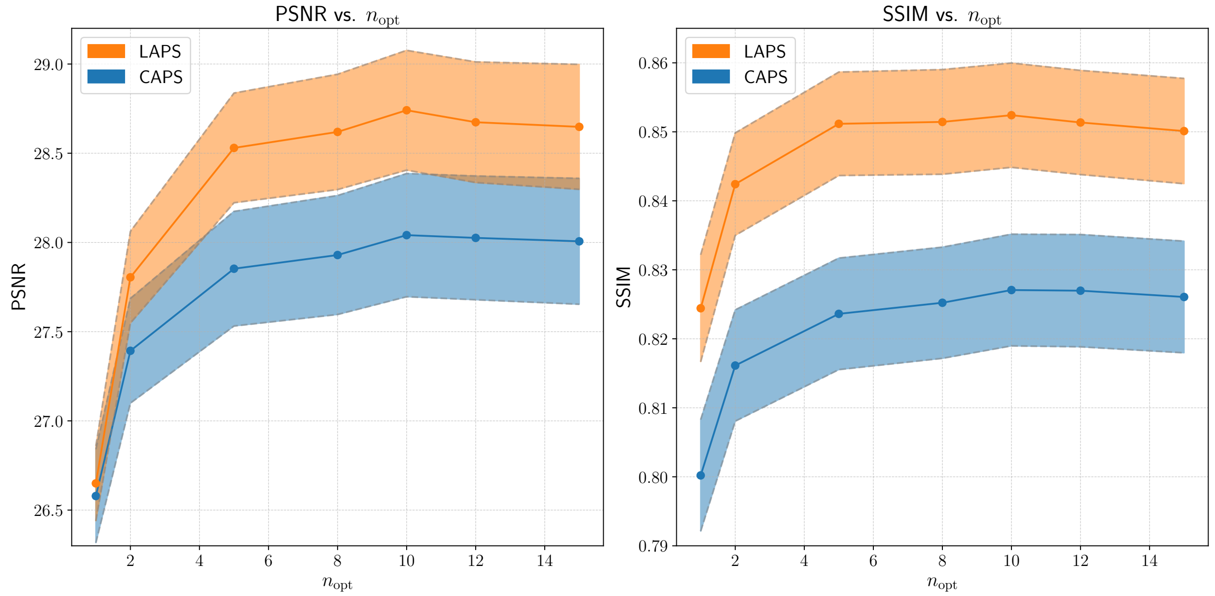

FIGURE S5 Performance of reconstruction metrics for various n_{opt}, with t_p chosen via `AutoInit`, for $n_{\text{step}} = 100$ DDIM steps. $n_{\text{opt}} = 1$ equates to sampling with PLDS[42]; we observe that for both CAPS and LAPS, $n_{\text{opt}} = 10$ maximizes PSNR and SSIM in the reconstruction test set. Additionally, LAPS consistently boosts performance compared to CAPS for each value of n_{opt}, showing the utility of the longitudinal prior.

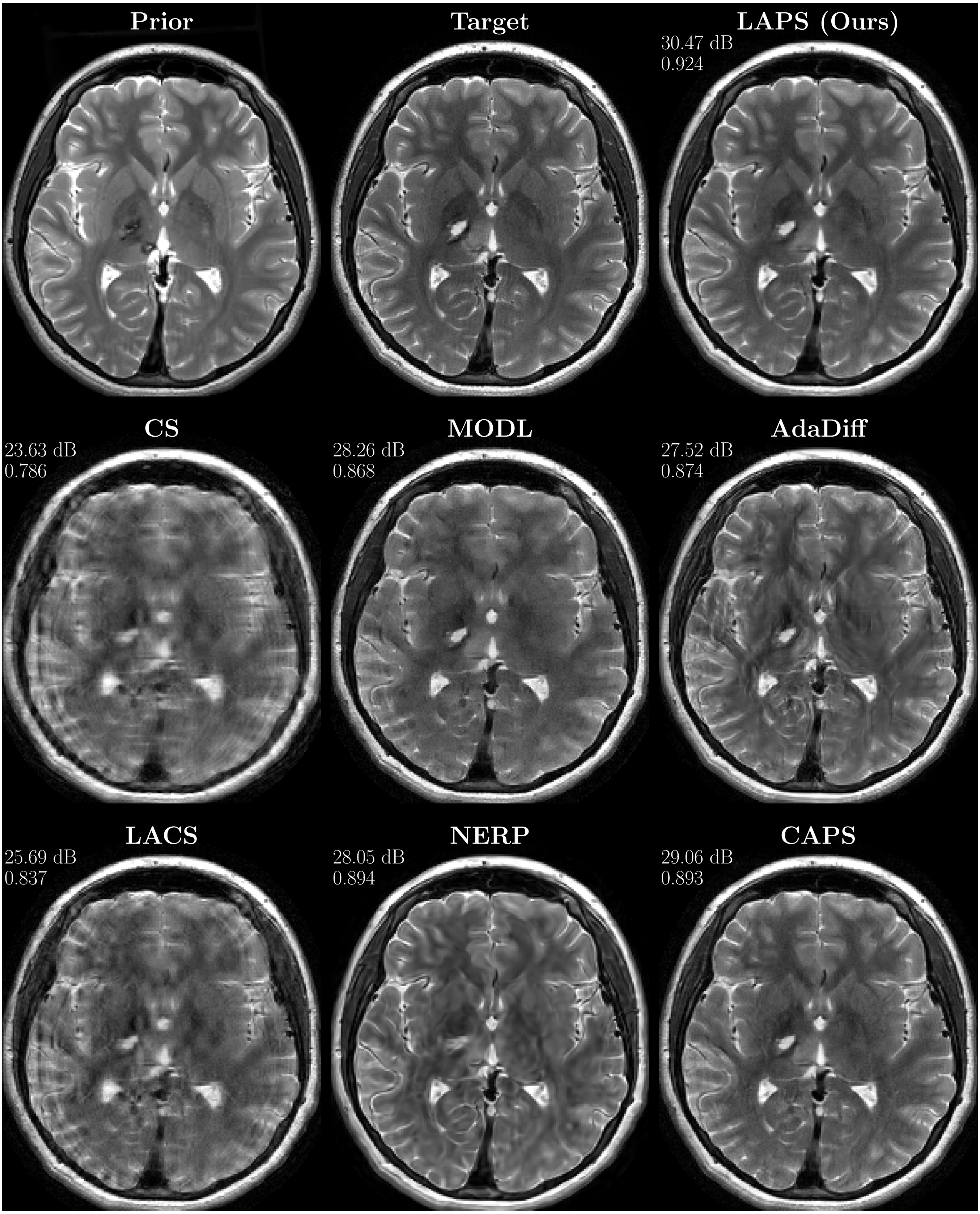

FIGURE S6 Additional reconstruction example for all methods at R=7 with 1D undersampling, with PSNR and SSIM shown to the left of each image, shown as an axial view for the large change coronal example in Fig. 6.

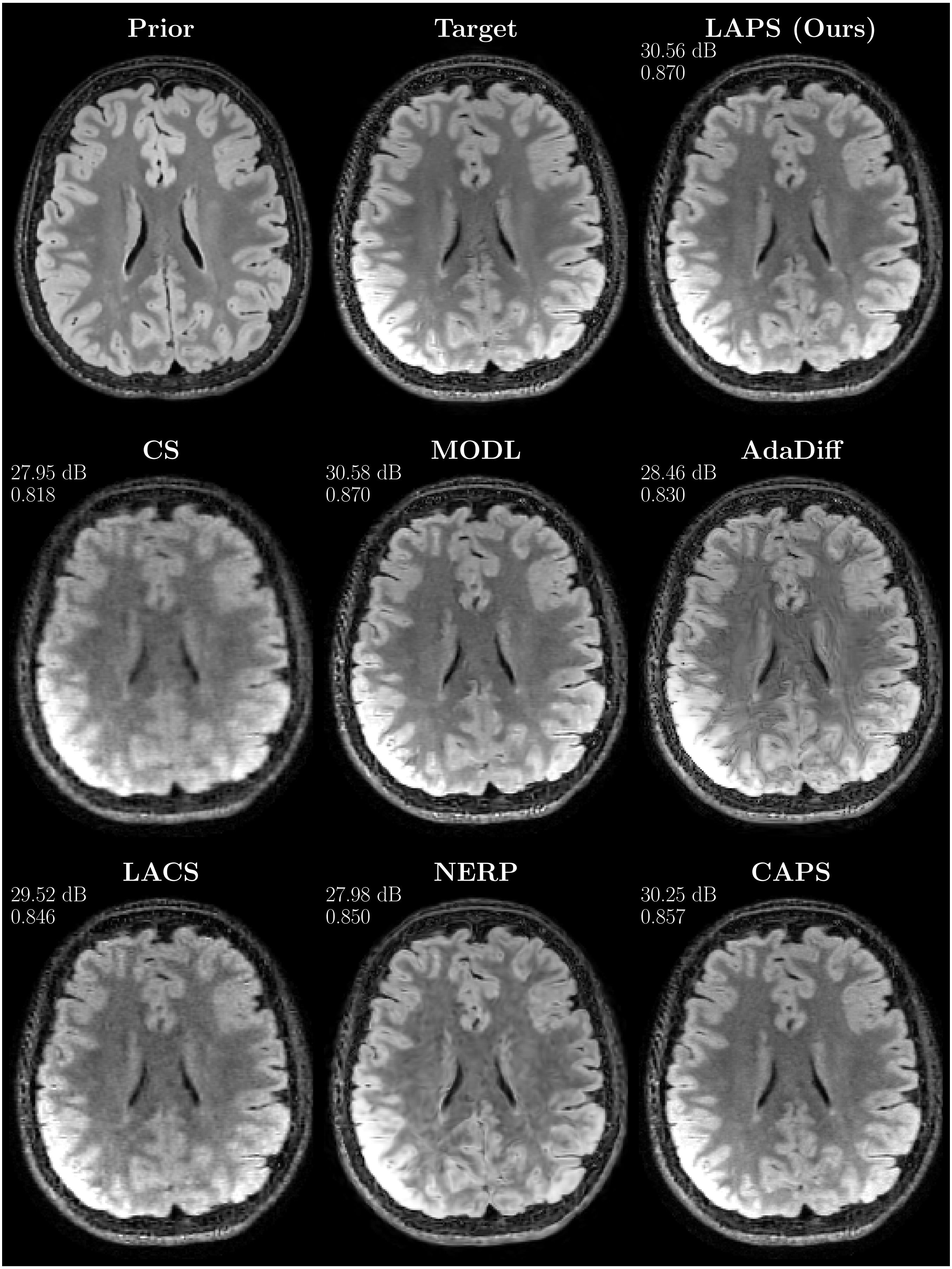

FIGURE S7 Additional reconstruction example for all methods at R=20 with 2D undersampling, with PSNR and SSIM shown to the left of each image, for a T_2-FLAIR contrast.

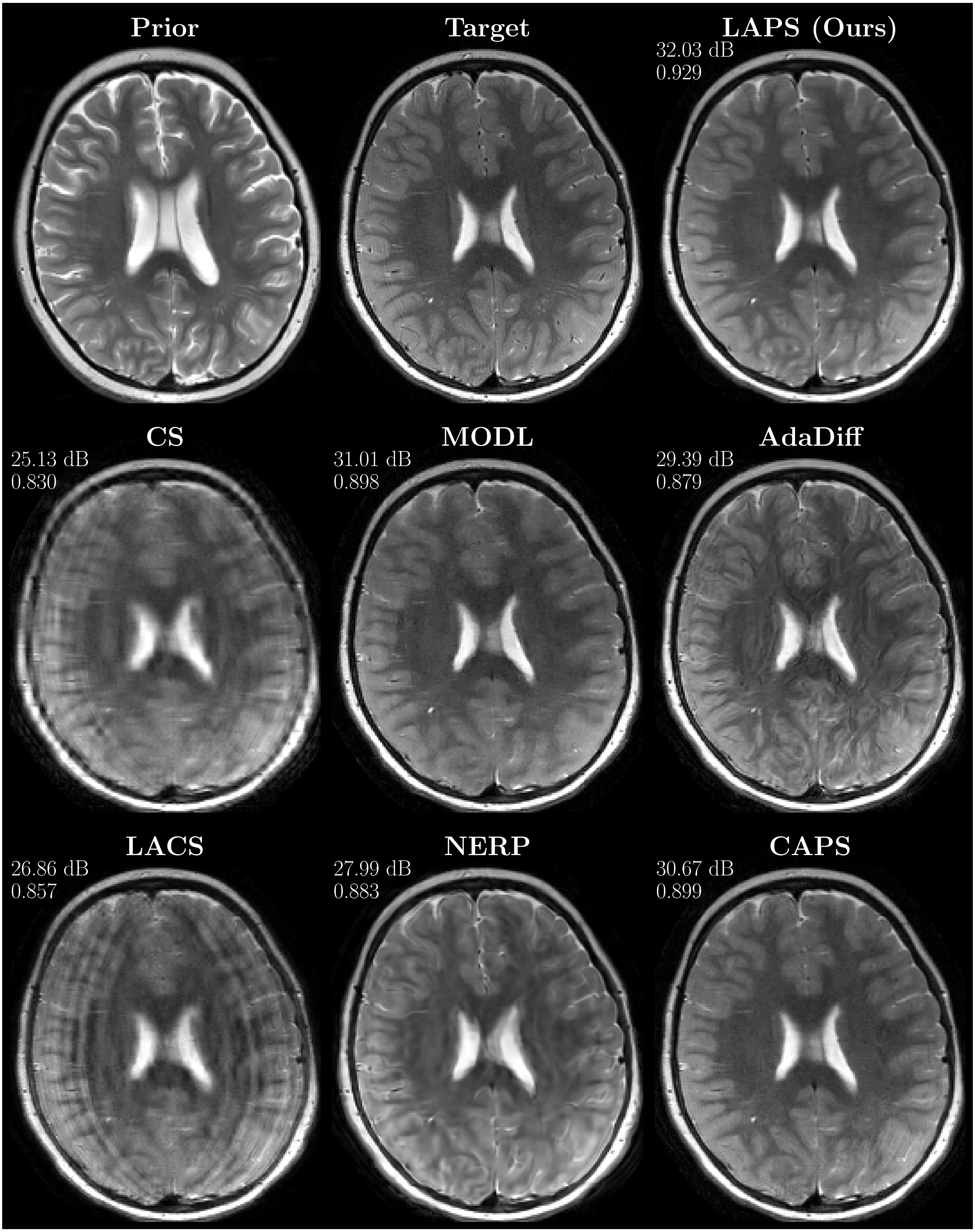

FIGURE S8 Additional reconstruction example for all methods at R=7 with 1D undersampling, with PSNR and SSIM shown to the left of each image, where the prior contrast slightly differs from the new scan contrast.
